# The Food Environments of Fruit and Vegetable Consumption in East and Southeast Asia: A Systematic Review

**DOI:** 10.3390/nu13010148

**Published:** 2021-01-04

**Authors:** Jason Tsz Him Cheung, Johnson Lok, Stuart Gietel-Basten, Keumseok Koh

**Affiliations:** 1 Division of Social Science, The Hong Kong University of Science and Technology, Kowloon, Hong Kong, China; thcheungaq@connect.ust.hk; 2 Institute of Public Health and Clinical Nutrition, Faculty of Health Science, The University of Eastern Finland, 70210 Kuopio, Finland; johnson.lok@uef.fi; 3 Division of Public Policy, The Hong Kong University of Science and Technology, Kowloon, Hong Kong, China; sgb@ust.hk; 4 Department of Geography, Faculty of Social Science, The University of Hong Kong, Pokfulam, Hong Kong, China

**Keywords:** fruit and vegetable (FV) consumption, food businesses, food environments, Asia

## Abstract

Fruit and vegetable (FV) consumption benefits the health of populations. This can be especially the case in locations which have undergone significant changes in their food environments, such as East and Southeast Asian countries. This current systematic review is the first to study the food environments—facilitators, barriers, and moderators—associated with FV consumption in East and Southeast Asia. We consulted five electronic academic databases of English peer-reviewed papers published between 2010 and 2020 and found 31 studies. Results of these studies show that individuals strongly perceive FVs as being high-quality and safe, and having trust in their benefits. Food businesses with modernized systems have significantly fostered the consumption of FVs. A main barrier to FV consumption, however, is financial concerns, exacerbated by food businesses with FV unavailability and urbanization-induced FV price inflation and dietary patterns. Demographics and shopping patterns further hinder FV consumption. The fragmented and conditionalized findings of the 31 studies require standardized FV consumption measurements. Unlike the impact of FV consumption determinants and their interactions in Western countries, those in Asia, particularly countries other than China, have been substantially understudied. Therefore, as the research gaps in studies of food environments and FV consumption in East and Southeast Asia urgently demand scholarly attention, this paper proposes recommendations that favour the consumption of FVs.

## 1. Introduction

Regular consumption of adequate amounts of fruit and vegetables (FVs) could reduce the risks of mortality from all causes, mainly cardiovascular disease, chronic diseases, and cancer [1,2,3,4]. It was estimated that 5.6 and 7.8 million premature deaths in the world in 2013 could have been attributable to an FV intake below 500 g and below 800 g per day, respectively [3]. Public policy interventions on a community level, such as changing the food environment, have shown more potential than dietary interventions on an individual level to promote FV consumption and subsequently a considerable reduction of diseases attributable to insufficient FV intake [5]. Evidence shows that not only individuals but also social and physical environments influence food choice [6]. It has been a longstanding notion that food environments are critical to the health of populations and should demand scholarly attention [7,8].

The concept of food environments has evolved over time. Food environments have been defined as the physical, social, economic, cultural, and political factors that impact the accessibility, availability, and adequacy of food within a community or region [9]. While the concept of food environments has traditionally been limited to the proximity of certain food outlets and the availability of food within markets, it has been extended to include other factors such as online food stores and nutrition information, the results of advanced technology and the rise of living standards. Food businesses, the settings, and the interactions between consumers and food environments have all been continuously shaping the food system, which contributes to certain dietary outcomes and requires comprehensive and recurring scrutinization. This paper reviews such interactions from three perspectives: individuals, food businesses, and environments.

In the last decade, studies have reviewed the relationship between food environments and diet and/or dietary outcomes in an effort to inform policymakers. For instance, Roy et al. reviewed the associations between the food environment and dietary behaviour of young adults in tertiary education settings in the United States (U.S.) and Europe [10]. Similarly, Engler-Stringer et al. reviewed the associations between food environment and children’s diets primarily in the U.S. and Europe [8]. One specific topic pertained to ‘food deserts’ and obesity, particularly common in Western countries such as the U.S. [11]. Holsten, mainly focusing on the U.S. and Australia [12], reviewed the relationships between obesity and community food environments, as did Gamba et al., who focused specifically on the U.S. [13]. Likewise, Cobb et al. reviewed the relationship between the U.S. and Canada local food environment and obesity [14]. While the examination of FV consumption among populations has gained popularity in recent years, the number of studies focusing on the impacts of food environments on FV consumption, primarily conducted in Western countries, has been increasing. Some researchers have examined diverse demographic subgroups such as children in the U.S., adolescents in the Netherlands, and three-to-five-year-old children in Australia [15,16,17], and others have studied the rural elderly in the U.S. [18].

Although the number of studies pertaining to food environments and FV consumption has increased substantially, this paper aims to fill two noteworthy research gaps. Only a limited number of reviews have discussed the overall interaction between adult consumers and food environments, and most reviews about FV consumption have targeted only specific demographic subgroups (e.g., children and adolescents). As consumers interact with the food environment on a daily basis, an exploration of how consumers perceive and respond to food environments would significantly enhance our understanding of this interaction and contribute to knowledge that would help shape public policy. Another gap in the literature relates to the focus of the majority of reviews, that is, one that targets Western countries, especially in North America and Europe. Few studies have examined the uniqueness of the Asian food environment and its impact on diet/dietary outcomes.

Rapid industrialization since the 1980s has resulted in unprecedented economic growth in East and Southeast Asia [19]. During such rapid economic growth and structural transformation, government policies in East and Southeast Asian countries have targeted the provision of food security; developed countries had already moved away from the agricultural sector that dominated market forces concerning the food supply [20]. East and Southeast Asian countries still experience food security, and only a few have managed to develop a sustainable food system to tackle food security challenges faced by poor households by stabilizing food prices. Thus, these are some of the unique characteristics and developments that separate East and Southeast Asian countries from Western countries. Meanwhile, the economic growth that marked the onset of the transition to urbanization led to a huge demographic shift that profoundly influenced the health of populations in East and Southeast Asia [21]. Unfortunately, as food availability and food purchasing power have increased in these regions during the past two decades, urbanized populations have begun to experience negative dietary changes that pose health risks [22]. One change could involve FV consumption; nevertheless, no study has examined the impact of FV consumption on the health of the overall adult population.

Despite the territory-specific heterogeneity of populations in East and Southeast Asia, they share common cultural patterns such as dietary habits [23]. For instance, with regard to FV consumption, consumers may buy vegetables at wet markets, and fruit vendors may import seasonal fruit from proximate countries. With the advancement of nutrition epidemiology and the initiative of fostering regional public health collaboration in Asia, a review of studies on FV consumption in East and Southeast Asian countries can produce insights into how stakeholders in the region can build food environments favouring FV consumption.

Therefore, this article aims to be the first systematic review studying the determinants associated with FV consumption in East and Southeast Asia. This effort adds value to the initiative addressing the importance of regional collaboration on promoting wellness in the population. The primary outcomes of this review are as follows: (i) a presentation of the results and implications of the included studies conducted in East and Southeast Asia, (ii) the identification of the facilitators, barriers, and moderators of FV consumption in food environments, and (iii) recommendations for policymakers and stakeholders on how to promote FV consumption.

## 2. Materials and Methods

This systematic review adhered to the PRISMA guidelines and checklist [24]. We conducted two searches in five electronic databases (the Web of Science, PubMed, Scopus, the Cochrane Library and CAB), dated 15 May 2020 and 17 May 2020. We did not use Google Scholar because of its overwhelmingly high sensitivity and lack of specificity. A professional information specialist with experience in systematic reviews conducted the searches and identified all of the articles with abstracts mentioning four key terms: (1) food environment, (2) fruit and vegetable, (3) East Asia, and (4) Southeast Asia. The review included studies conducted in the following territories: China, Japan, Malaysia, South Korea, Taiwan, Thailand, and Vietnam. Throughout history, these countries have comprised the East Asian cultural sphere (i.e., China, Japan, Malaysia, South Korea, Taiwan (In this analysis, Taiwan is considered separately from China. No political statement is intended.), Vietnam) or a territory strongly influenced by China (i.e., Malaysia, Thailand). We excluded other countries because of their rudimentary development (i.e., Cambodia, Laos), extreme landscape (i.e., Mongolia, Indonesia, the Philippines), aberrant political system (i.e., North Korea), and unique religious food culture (i.e., Brunei, Indonesia). The terms used for each database search in this review appear in Appendix A. After the official search, we conducted forward and backward citation searches and identified 8469 articles from the five databases and Cochrane Controlled Trials, listed in Table 1.

The major factors of ‘food environment’ that impact the accessibility, availability, and adequacy of food within a community include physical, social, economic, cultural, and political factors, which we classified as relevant for inclusion in this study. We excluded studies if the research topics focused heavily on agriculture or business (e.g., the logistics of FV, a low-carbon environment, and pure business modelling of FV sales). For ‘study population’, we excluded studies solely examining subgroups of a population (e.g., adolescents, the elderly, children in households, individuals with diseases, medical students at a university, school canteen users, and students). The reasons for exclusion were twofold: (i) the focus of this review is overall interactions of FV consumers with food environments in the community, and (ii) presenting all population types and variables in a paper is unfeasible. We included only studies that took place in or partially in East Asia and/or Southeast Asia and only English peer-reviewed articles published in 2010 or after. We also included studies that explored the accessibility, availability, intake, perceptions, and purchase of FVs, and the preference and willingness to pay for FVs. Excluded studies were those that targeted specific chemicals or attributes of FVs (e.g., toxigenic fungi and trace elements of Chinese cabbage) and conference papers, letters to the editor, posters, reviews, symposium papers, systematic reviews, and meta-analyses.

**Figure 1:**
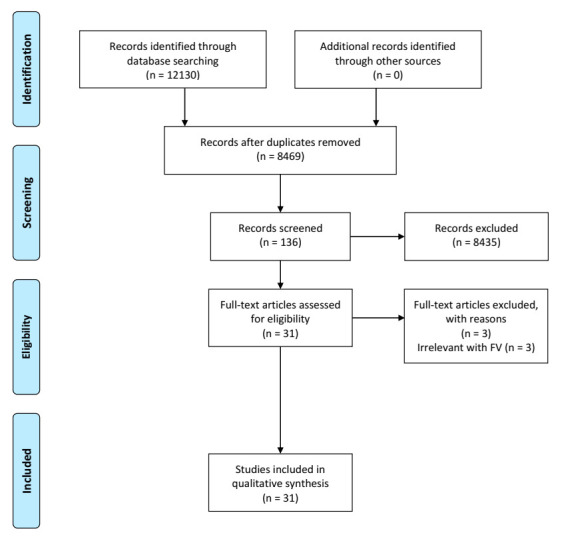
Article selection process.

After the database searches, two reviewers (J.T.H.C. and J.L.) downloaded, imported, and merged the selected studies in an electronic reference manager. Then, using the Rayyan QCRI tool (Qatar Computing Research Institute (Data Analytics), Doha, Qatar), reviewers conducted the screening of the 8469 abstracts after removing duplicates [25] and imported the merged records with the blind function on so that studies can be included or excluded without mutual influence. Following the eligibility criteria, reviewers chose ‘include’, ‘maybe’, and ‘exclude’ for the application of each abstract. After the initial screening, with the blind function turned off, reviewers evaluated the inconsistent inclusion, exclusion, and ‘maybe’ articles and scheduled discussions with each other about whether articles should be included in the study until they reached a consensus. Finally, reviewers assessed the full texts of each article for final inclusion. See Figure 1 for the overall selection process.

For the sensitivity analysis, reviewers conducted a quality evaluation of studies using a tool developed by Behera et al. [26] and evaluated five aspects of each study: presence of evidence analyzed quantitatively or qualitatively, reports of both added value and limitations of the study, justifiability of the outcomes (any involvement of techniques), reliability and recognizability of the publication source (Citations no. + H-Index), and evidence of a comparison of the methodologies used in the study and other methodologies. The protocol for assessing the five aspects is shown in Table 2. According to the protocol, the highest quality score is 9, and studies with quality score 4.5 or lower fall into the exclusion criteria. The journal of publication, the number of citations, the H-index, and the final quality evaluation score of each study are listed in Table S1. Reviewers appraised the quality of the included studies by performing the stated protocols independently and achieved acceptable Cohen’s kappa inter-rater reliability (*κ* = 0.87; *p* < 0.01).

From each eligible study, we extracted the following data: the author(s), year of publication, study design, territory, and population, sample size, studied food environment(s), categories of FV consumption, and key reported findings. We then analyzed the findings from three perspectives: individuals, food businesses, and environments.

## 3. Results

The search yielded 8469 articles after the removal of duplicates. By screening abstracts and titles, we excluded 8435 studies that did not meet the eligibility criteria and after screening of the full texts, we excluded three additional studies irrelevant to FV consumption. Thus, 31 studies, all of which passed the quality evaluation, remained for inclusion in this review.

**Figure 2:**
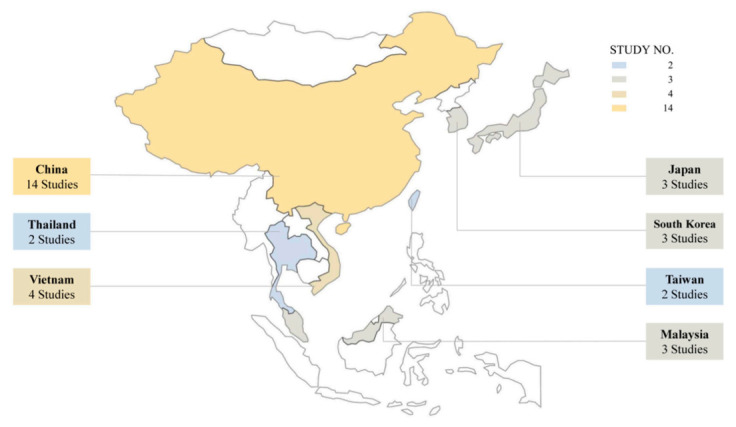
The number of studies in each territory included in the review. Note: Mongolia, Brunei, Cambodia, Indonesia, Laos, North Korea, and the Philippines were excluded from this review.

Using the Free and Open Source, QGIS (version 3.10.4) (QGIS.org), we plotted the number of eligible studies from each territory on a map of East and Southeast Asia (Figure 2). China had the highest number of eligible studies (14), followed by Vietnam (4), Japan (3), Malaysia (3), South Korea (3), Taiwan (2), and Thailand (2). The number of studies in each territory suggests two main implications. One is that a predominantly high number of studies examined the impacts of food environments on FV consumption in China, possibly attributable to the increasing prevalence of dietary health issues associated with its unprecedented economic progress and rapid nutrition transition stemming from the rise in the standard of living over the last decade [27]. In addition, as few studies took place in East and Southeast Asian countries other than China, the other implication is that these countries have a large research blind spot and possible lack of resources available for investigating how food environments affect FV consumption.

**Figure 3:**
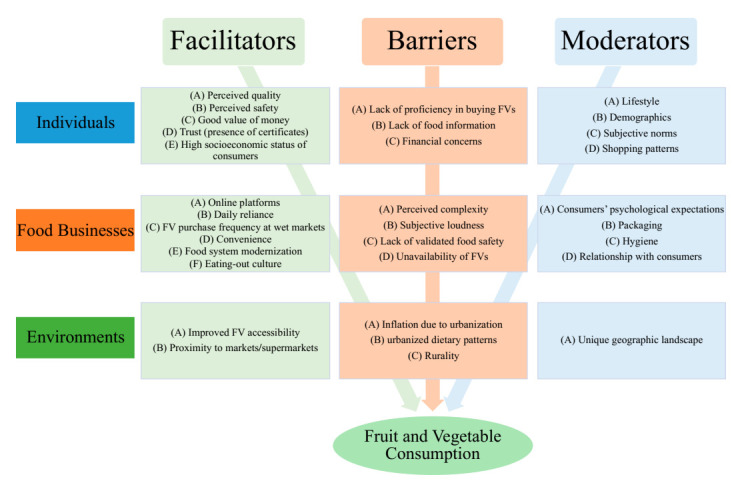
The overview of the facilitators, barriers, and moderators of FV consumption.

**Figure 4:**
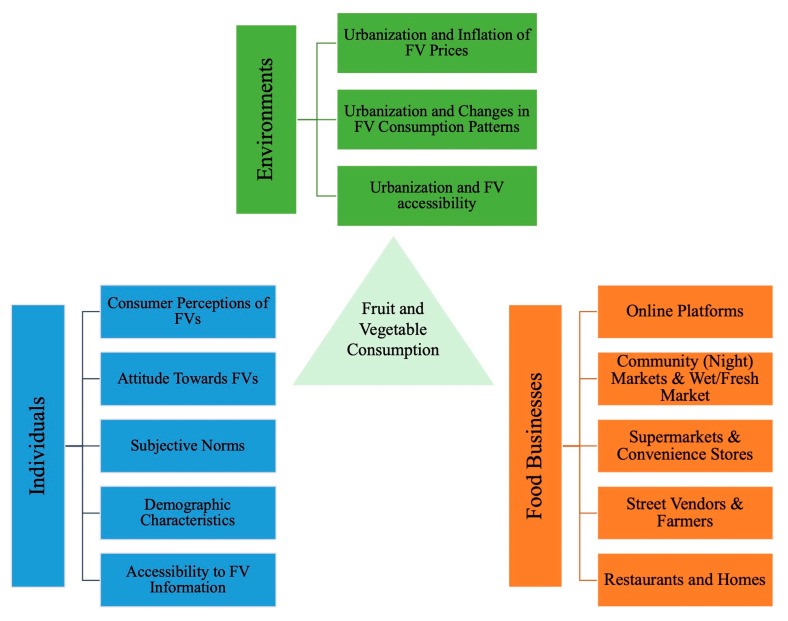
The interactions of FV consumption in three perspectives: individuals, food businesses and environments.

This review analyzed the 31 studies by systematically categorizing various determinants into facilitators (18 studies), barriers (18 studies) and moderators (15 studies), as shown in Table 3, Table 4 and Table 5. The overview of the facilitators, barriers, and moderators of FV consumption is shown in Figure 3. The determinants are discussed from three perspectives: individuals, food businesses, and environments. The interactions of FV consumption in each perspective are illustrated in Figure 4.

### 3.1. Individuals

#### 3.1.1. Consumer Perceptions of FVs

Consumers perceived product attributes, particularly freshness, to be significantly associated with their decision to purchase FVs [28,42,44]. Consumers agreed that nutrition was a strong criterion for ‘good quality’ [28]. Consumers also preferred higher levels of vitamins when they purchased fruit [39]. Other variables associated with their purchases of FVs included cleanliness, colour, taste, packaging, smell, and involvement in religious processes [28]. Consumers were reportedly more likely to purchase normally shaped FVs than moderately or extremely abnormally-shaped FVs [35]. Environmental concerns and social trust embedded in consumers, however, may have driven consumer purchase intentions towards abnormally-shaped FVs. The environmental sustainability of the FVs was another interesting variable [39]. We found that consumers preferred fruit that involved greater reductions in carbon emissions, more efficient use of water resources, and less waste/packaging.

A highly addressed assessment criterion of FV was food safety, indicated by a lack of chemical residues, pesticides, fertilizers, and preservatives [28,40,43]. Interestingly, consumers at wet markets considered vegetables grown within the city boundaries as safe [43].

Another attribute influencing FV purchase decisions was price. Interestingly, although consumers’ definition of ‘good quality’ included the criterion of good value for the money [28], consumers did not prefer FVs at a higher price [28,30,38]. Targeting the negative influence of price on FV purchase behaviours, one study reported that financial incentives could encourage consumers to purchase FVs [36]. Unlike the dominant findings, one study reported that price was not associated with Chinese consumers’ decisions to purchase imported fruit, which they perceived as high quality [56]. In general, however, the findings suggested the heterogeneity of consumer perceptions of the importance of price in FV purchases. Further study could investigate such heterogeneity.

#### 3.1.2. Attitude Towards FVs

There is a significant association between the attitudes and intentions of consumers purchasing FVs throughout the years. One of the most addressed factors was their trust in the source of FVs. Studies found that consumers tended to purchase FVs from their most trusted FV providers [29] and FV products with official food safety certifications, brands, or labels [30,43,44]. Specifically, a study reported that consumers preferred proprietary enterprise brands over cooperative brands [45].

Another variable related to consumer attitudes towards general FVs was their food shopping patterns. That is, shoppers who had a frequent habit of buying FVs tended to mention the importance of food safety and quality more than inexperienced FV shoppers [29]. This finding demonstrates the effect of consumers’ familiarity with the FV itself or the process of buying FV on their attitudes toward purchasing FVs. Nevertheless, consumers often habitually go to their favourite sites or locations for their daily fresh FV shopping once it becomes an everyday practice [43]. That is, consumers did not seek alternative food vendors outside of their ‘comfort zones’. This finding highlights the effects of common FV consumption patterns, despite consumer proficiency in FV purchasing. Contrary to these findings, one study reported no clear relationship between FV consumption levels and food shopping patterns [32]. This finding sheds light on a potential research gap in the relationship between consumers’ FV shopping patterns and their dietary outcomes. More studies are needed to examine the assumption of a linear relationship between purchasing FV and eating FV.

Similar to the studies showing that the status of organic FVs may differ from that of non-organic FVs, our review concluded that consumer attitudes towards organic FVs were unique, which demands further study. We found that one’s daily dietary lifestyle was associated with the purchase and consumption of organic FVs, the driving factor of which was the desire to try new organic products and pursue a healthier lifestyle [49]. Similar to consumers who purchased non-organic FVs, those who purchased organic FVs preferred fruit with higher environmental sustainability [38]. These findings suggest that consumers are driven by a personal lifestyle influenced by environmental awareness. 

Compared to consumers of non-organic FVs, consumers of organic FVs were more aware of the price and quality of organic FVs [40]. In fact, higher prices of the organic FVs had an association with consumers’ unwillingness to purchase certified organic vegetables, suggesting that despite their perceptions that organic FVs were high quality, price was the main determinant of their decisions to purchase organic FVs.

In addition to price and quality, certification also had an association with the consumption of organic FVs [40,43,45]. More specifically, consumers preferred and trusted certification by non-governmental organizations (NGOs) and the European Union (EU) over stringent green and government certification labels (e.g., ChinaOrg) [40,45], so they were willing to pay higher prices for certified organic FVs [40]. Interestingly, although the presence of certification influenced the purchase of organic FVs, consumers generally did not have the intention to actively engage in searching for alternatives that would guarantee food safety via official certification [43]. One interesting finding underscored the importance of the traceability of organic products, that is, information about the origins of farms and sites where FVs are processed, circulated, and marketed [40].

#### 3.1.3. Subjective Norms

With regard to the shopping perceptions of consumers, their subjective norms related to FVs strongly determined their purchasing behaviours. One study that focused on the association between the ethnocentric tendencies of consumers and their purchasing of imported fruits [56] found little association between the two. Interestingly, the only statistically significant finding was demographic in nature: that respondents who were older than 25 years and those who held less than a bachelor’s degree tended to be influenced by ethnocentrism in their purchase behaviours of imported fruits. This finding suggests that when it comes to FV purchasing behaviour, ethnocentrism plays a greater role among older demographic groups with less education.

Another dimension of subjective norms pertains to consumer lifestyle. One study identified three lifestyle groups: (i) risk takers (i.e., individuals who are self-centred, spontaneous, self-indulgent, and success-driven; (ii) experiencers (i.e., individuals who seek variety and novelty and embrace challenges and risks); and (iii) traditionalists (individuals who are traditional and conventional) [56]. None of the three lifestyle groups had a statistically significant impact on the sensory or non-sensory purchase attitudes of either domestic or imported fruits. Sensory attributes denote the taste, fragrance, safety, and freshness of fruit, and non-sensory attributes denote the price and brands of fruit. Interestingly, the study found statistically significant relationships among risk takers and traditionalists concerning an intention to purchase imported (U.S.) fruit.

In addition to ethnocentrism and lifestyle differences, universalism was a focus of a study [57] that found a significant positive correlation between universalism and attitudes towards buying vegetables, particularly the relationship between consumer attitudes towards organic vegetables and the extent to which consumers rated universalism. Studies reported that a values-attitude-behaviour hierarchy was present among Chinese consumers on organic vegetables and their choices of environmentally-friendly organic vegetables stemming from a universalist attitude, prevalent in Western countries. It has been suggested that universalism is a norm that dominates consumer FV purchasing behaviours.

#### 3.1.4. Demographic Characteristics

The majority of studies reported significant relationships between demographics and FV consumption. In terms of socio-economic status, there were associations between higher consumption levels of fruit and higher incomes, better education, and urban residency [38]. Surprisingly, there was a negative correlation between the education variable and the consumption of vegetables. When purchasing FVs, consumers with higher education levels preferred freshness and safety over taste [42]. Interestingly, even though the proportion of individuals in Seoul, Korea with an insufficient food supply stemming from financial hardship had significantly decreased over time, one study found that a growing number of its people were consuming FVs less than once per day [51]. Therefore, dissecting the various underlying factors that shape the ever-changing interactions between demographics and FV consumption calls for extended longitudinal study.

Another demographic variable found to have an association with FV consumption was age. Senior FV consumers tended to be more concerned about the food safety of vegetables [29]. With regard to food consumption patterns, there was an association between young people and limited consumption of FVs while older people suffered from an insufficiency of FVs because of financial difficulties [51]. One study found a significant relationship between FV consumption and meal types [34]. While the consumption of vegetables during breakfast, in general and specifically among adults aged 19 to 49, declined, it increased during lunch. One interesting finding was an increase in vegetable consumption during snack time among the young adult population. One study found no significant difference between vegetable consumption during either lunch or dinner among people who were over 50. These findings suggest the importance of tailored interventions related to meal types for different age groups.

We identified two types of consumers with diverse intentions to purchase FVs online: (i) the ‘online-food conservative’, who has weak intentions to purchase FVs online, and (ii) the ‘online-food-pioneer’, who has strong intentions to purchase FVs online [41]. The findings showed that the ‘online-food-conservative’ was normally low income, unmarried, and younger than 30 or older than 40, and held a medium or low-level job position (e.g., students); and the findings showed that the ‘online-food-pioneer’ was normally medium or high income, married, and aged 31 to 40, and held a high-level or a self-employed position. These findings suggest that attitudes towards online FV shopping may be strongly associated with the demographic and socio-economic status of consumers.

Studies also showed that in addition to socio-economic status and age, there was an association between one’s sex and FV consumption. They found that urban women had stronger intentions to buy organic FVs [33] than rural women and that women tended to show more concern about the safety of vegetables than men [29]. To date, however, no study has focused on the consumption of FVs by men.

#### 3.1.5. Accessibility to FV Information

Although consumers generally differed with regard to how they obtained information about FVs, we found that in general, they had received little knowledge about FVs, which contributed to low FV consumption. Consumer uncertainty about whether or not to try or buy organic vegetables associated with a lack of knowledge about the prices and quality of organic vegetables [49]. This finding further confirmed the close relationship between product choices and access to information about certain vegetables, such as the origin of the vegetables [43], reflected by consumers’ desire to know if a vegetable was grown in the vicinity. There was also an association between consumer confidence in purchasing FVs and a past experience with food-borne illnesses, suggesting that lack of information about how to select, process, and clean FVs would affect FV consumption. Not surprisingly, consumers in various demographic groups had diverse means of obtaining information about FVs [29]. Most consumers accessed such information via television and broadcasting [29]. Young, well-educated consumers, however, tend to search the Internet to do so. Interestingly, thanks to advancements in technology, an increasing trend of those searching online to buy organic FV has been reported [33]. The findings suggest the possible advantage of expanding FV advertising in online platforms. They also highlight the importance of delivering tailored food information to different demographic groups.

### 3.2. Food Businesses

#### 3.2.1. Online Platforms

Because of recent advancements in technology, the topic of ‘online shopping’ has prompted researchers to examine how online platforms interact with FV consumption. Interestingly, urban women had strong intentions to buy organic FVs online [33]. Female customers in urban areas perceived online shopping as fashionable, fun, cost-effective, time-saving, convenient, user-friendly, and amenable to price comparison, less expensive because of discounts, safe because of the presence of organic/certification labels and consumer liability insurance, and meaningful because of their concern for farmers. In other words, online platforms provided urban women with a humanized, safe internet interface and procedures that ensured the quality of their FV purchase and the safety of the transaction [33]. Therefore, attitudes towards buying organic agricultural FVs online could positively influence urban women’s purchase intentions.

Having examined the complexity of the online setting, several studies found that there was a positive association between consumer attitudes towards online food shopping and their online FV purchase intentions [41]. The studies focused on two innovation-adoption characteristics, perceived incentives and perceived complexity. Consumers with strong perceived incentives for online food shopping tended to have positive attitudes and purchase intentions. Those who had high perceived complexity had less tendency to purchase FVs online. Interestingly, the association between consumers’ perceived risk (e.g., entering into an insecure transaction and receiving a low-quality product) and their intention to purchase FVs online was not statistically significant. In addition, attitudes and perceived behavioural control (PBC) could predict one’s intention to buy organic FVs [33]. In other words, enhancement of consumer appreciation of the advantages of online platforms by various marketing strategies such as the keyword search method would increase the intention of consumers to buy FVs online. It should be noted, however, that the study was limited to only urban women. Therefore, further studies are needed to examine the relationship between the online shopping behaviours of other population types (e.g., men, elderly, disabled people, busy people) and their intention to purchase FVs.

#### 3.2.2. Community (Night) Markets

The findings regarding the relationship between community, or night, markets and FV consumption were unique. There was an association between consumers’ FV purchase decisions at night markets and the perceived quality, freshness, and safety of fresh FVs [30]. Even though vegetables in the community markets were generally cheaper because of the relatively loose packaging or pre-washing [38], consumers tended to rely on community markets as their second choice if they had access to supermarkets. For open-air community markets, in addition, an increase in crowd density in open-air community markets showed a positive correlation with a decrease in acoustic comfort, which would in turn affect the purchase behaviours of FV [54]. The sound produced by plastic bags may cause auditory discomfort in consumers. As the subjective loudness in the FV sales area of the markets was higher than it was in other food areas, this finding suggests the importance of the urban design of open-air community markets in high-density cities in East and Southeast Asian countries.

#### 3.2.3. Wet/Fresh Market

Compared to community markets, wet/fresh markets were the focus of more studies. Consumers purchasing FVs at wet markets were more likely to consume a sufficient number of FVs, but the results were not significant [32]. This finding highlights the significance of the presence of wet/fresh markets in the community because consumers tend to rely on them in their daily FV purchases. Interestingly, contrary to the findings targeting community markets (night markets), no statistically significant association between consumer decisions to purchase fresh FVs at wet markets and their perceived quality, freshness, and safety [30]. Consumers aged 47 or less tended to show a concern for the safety of fresh FVs when they shopped at wet markets [30]. These findings could indicate that the psychological expectations of consumers at wet/fresh markets are lower and that younger demographic groups are still concerned about FV safety regardless of where they are sold. These findings call for further research on the effects of the perceptions of wet/fresh markets on the psychological expectations of consumers and their FV purchase behaviours. 

Interestingly, most consumers at wet/fresh markets were concerned about prices and their relationship with sellers [40]. Furthermore, as they did not have first-hand knowledge of food safety risks [43], they relied on their relationship with sellers to establish trust in their daily FV purchases. This finding suggests dominant continuity embedded in the personal relationships between consumers and FV providers at wet/fresh markets. 

#### 3.2.4. Supermarkets

Studies have comprehensively examined FV consumer behaviour in supermarket settings. Consumers with convenient access to supermarkets appeared to consume more fruit but fewer vegetables [38], a finding that highlights the importance of the proximity of supermarkets in communities. The prevalence of modern supermarkets improved not only the availability of food but also the standard of living, which boosted fruit consumption [31]. With regard to sales, consumers preferred supermarket vegetables because they were monitored by supermarket management systems, which provided consumers with a diverse assortment of vegetables from multiple suppliers [40]. Also, supermarkets provide quality certification labels, traceability information and health information which encouraged consumers to purchase vegetables [37,40]. These findings suggest that consumers generally hold multiple positive attitudes towards supermarkets, which leads to their increased consumption of FVs. Interestingly, consumers had contrasting views on whether supermarkets should sort out or continue to sell unattractive FVs that are 30% larger or smaller than average due to their concern about environmental waste [35]. Therefore, this interesting finding denotes the important role that supermarket marketing strategies could play in the psychological purchase behaviours of consumers.

Regarding the dissemination of food information by supermarkets, consumers addressed how they acquired food information that led to their decisions to purchase FVs. Specifically, one study found that point-of-purchase (POP) intervention strongly influenced consumer FV purchase decision making [37]. With POP intervention, consumers tend to make wiser decisions if they are provided health-related food information. The study found that there is a positive association between such intervention on sales and FV sales. In addition, the study found that POP information could mitigate the decline of FV consumption during the fall and winter seasons due to the seasonality of the provision of certain FVs by providing recipes on the cooking and preparation of seasonal and non-seasonal FVs. However, in general, few studies explore other means of disseminating food information in different settings, which calls for further study on food information dissemination.

#### 3.2.5. Street Vendors

Like the various types of markets, street vendors play an important role in providing fresh FVs [43]. However, very little research has been devoted to the study of street vendors. Interestingly, we found that informal street vendors expressed absolute confidence in the safety of vegetables even though no evidence was available to validate this belief. This finding could explain why consumers were less likely to purchase FVs from street vendors than formal markets/supermarkets.

#### 3.2.6. Convenience Stores

One study examined FV consumption in relation to convenience store lunch boxes [52]. The study reported that the average number of vegetables in lunch boxes was fewer than one serving size (~70 g), which falls below the recommendation of two to three servings of vegetables per meal. It also found that vegetables were cooked by mostly stir-frying and pan-frying in oil, indicating deficiencies in the diversity of cooking methods, which could influence consumer intake of FVs. Even though convenience stores, unlike cafeterias and restaurants, are conventionally not classified as normal meal providers, the findings above provide useful information about how convenience stores contribute to consumer FV consumption.

#### 3.2.7. Restaurants and Homes

Regarding restaurants and homes, consumer intake of vegetables at cafeterias and restaurants increased over time for all ages [34]. Specifically, seniors over 65 consumed significantly more vegetables at restaurants, but adults between 19 and 49 consumed fewer vegetables at home. Individuals between 16 and 64 showed a trend of decreased vegetable consumption at home. The findings suggest that most demographic groups tended to be eating outside the home more often over time, which indicated greater vegetable consumption.

#### 3.2.8. Farmers

The availability of FVs played an important role in determining the FV purchasing behaviours of consumers [40]. Consumers, in general, were willing to pay the price for increased availability of certain FVs, especially the certified FVs, in supermarkets. One unique factor influencing FV availability was the seasonality of FVs [37]. Typically, as the production, distribution, and sales of FVs decrease during the fall and winter seasons, policymakers should introduce specific remedies to encourage farmers to secure FV availability.

### 3.3. Environments

#### 3.3.1. Urbanization and Inflation of FV Prices

Studies have found that FVs sold at higher prices in urban locations [31,46,55]. In fact, a 10% increase in urbanization raised the price of vegetables by 0.9% and fruit by 0.7% [31]. They also reported that 86.7% of the population in low-income urban communities consumed FVs fewer than five times per day, which was below FV intake guidelines [46]. As the prices of FVs have been an indicator of FV consumption, researchers have examined the extent of the impact of urbanization on FV prices.

#### 3.3.2. Urbanization and Changes in FV Consumption Patterns

One study found a change in the pattern of FV consumption: Urban dwellers consumed more fruit and less grain-based food, possibly the result of the higher standard of living that followed urbanization [48]. The study also reported that the increase in FV consumption was faster in urban areas, with the urban population preferring FVs instead of rice, regardless of their urban status and income [47]. The demand for high-value food such as FVs increased significantly, as urban consumers consumed 114% more fruit than their rural counterparts [38]. Another study, however, identified a discrepancy between fruit and vegetable consumption by suggesting that urbanization reduced the demand for vegetables while increasing the demand for fruit [31]. This interesting finding invites additional research on the soundness of the negative effect of urbanization on vegetable demand.

In the rural community food environment, one study reported that the consumption of vegetables was significantly low while that of fruit and related products underwent a rapid increase [50]. This finding challenged the conventional notion of rural residents self-supplying FVs for their daily food needs. Thus, further study regarding the consumption patterns of FVs in rural areas should be encouraged.

#### 3.3.3. Urbanization and FV Accessibility

Urbanization improved FV accessibility via the establishment of more modern supermarkets [31]. Rising living standards offered by urban food businesses led to an increase in the consumption of fruit. With the improved availability of FVs and accessibility of FV providers, consumers could access a diverse assortment of FVs easily. Despite the closer proximity of supermarkets in urban areas, which facilitated fruit consumption [38], it took longer for those living in the least wealthy neighbourhoods to access supermarkets and vegetable and fruit stores [53]. This finding was consistent with previous studies that took place in Western countries regarding the effects of socioeconomic status on accessibility to food stores [5]. This finding could be explained by low-income consumers’ inability to afford the rent for properties near food businesses. However, one study found contradictory evidence of the benefits of closer proximity to markets/supermarkets stemming from urbanization [42], reporting that consumers did not place much importance on the geographic location or landscape of FV providers, possibly attributed to the unique geography in which FVs were grown and available throughout Taiwan, where consumers normally had access to a diverse assortment of FVs.

Specifically, for organic FV, there is an association between organic FV buying experience in an urban setting and organic FV purchase intentions [33,49]. Although very few consumers had had experience buying organic FV [49], the majority expressed intentions to buy organic FVs if they were able to find them in common FV places where they are sold, such as the markets. This finding suggests that consumers encountered difficulties accessing organic FVs. As the study was conducted in Hanoi, which has been developing rapidly, the finding implied the importance of improving accessibility to organic FVs in the urban planning of developing cities. 

The impact of the rural community food environment on FV consumption in terms of accessibility to FVs differed from that found by studies in an urban setting. Rural individuals with greater access to food markets did not consume as many FVs as those with less access to food markets [50]. One explanation for this finding is that those living close to markets substituted FVs with processed food sold in modern food markets while those living far from modern food markets consumed more FVs, attributable to their planting of FVs in more spacious yards.

## 4. Recommendations

**Figure 5:**
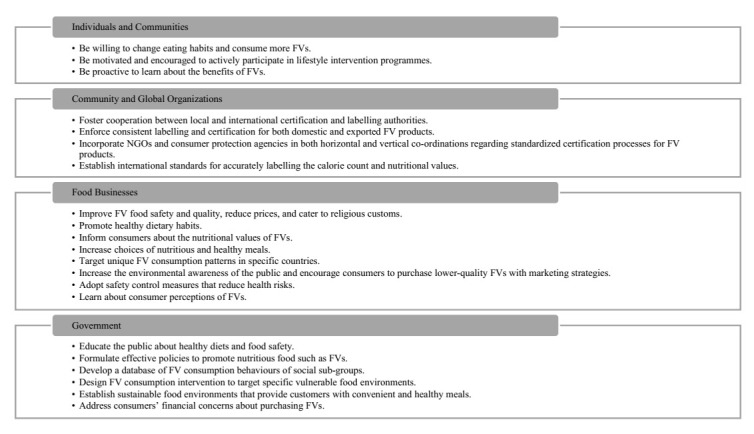
Recommendations for different stakeholders and policymakers to improve FV consumption.

While studies commonly reported findings on the associations between a number of variables and FV consumption, scholars should attempt to find ways to increase the consumption of FVs. As Asian countries arguably share similar food cultures, they should foster knowledge exchange and promulgate realistic policies that target the interactions between FV consumption and food environments. Upon reviewing the papers included in this paper, we propose the following recommendations that stakeholders and policymakers (i.e., individuals, communities, food businesses, governments, and local and global organizations) should consider to improve FV consumption. The overall recommendations are outlined in Figure 5.

### 4.1. Individuals and Communities

For individuals and communities, a willingness to change their eating habits to consume more FVs is crucial. In particular, we should motivate and encourage individuals to actively participate in lifestyle intervention programmes (e.g., the healthy plates programme) and receive information about the benefits of FVs through government public service announcements in the media. Awareness of the benefits of FV consumption is fundamental in shaping the behaviour of a population. We should advocate urban populations, especially those that are less proficient at using online platforms, to purchase FVs online. In addition, we should encourage rural populations that engage in agricultural activities to consume a portion of the FVs they grow.

### 4.2. Food Businesses

Food businesses should improve FV food safety and quality, specifically regarding freshness and cleanliness, reduce prices, and cater to religious customs, which can earn better reputations for markets and retailers and build consumer confidence. They also need to improve sophistication, openness, and responsiveness to the prices of FVs so that consumers have a greater willingness to buy high-value FV products. Businesses could promote healthy dietary habits by offering a choice of healthy meals and implementing interventions with low-value monetary incentives. Another way businesses could facilitate FV consumption is to inform consumers about the nutritional value of FVs and choices of nutritious and healthy meals by incorporating POP health information. 

To promote the sales of FVs, food businesses should adopt sales strategies that target unique FV activities in specific countries. For instance, they may partake in the potential establishment of organic food markets in their own localities. They could also increase awareness among generational cohorts and emphasize specific key FV product attributes that satisfy their demands and requirements. One way to achieve awareness is to appeal to the concerns of the public about the environment and promote a willingness to purchase lower-quality FVs (e.g., those with shape abnormalities). Online businesses should promote the purchase of FVs by including price comparison functions, discounts, easy-to-use shopping procedures, easily accessible interfaces, lower price, and quality control. These businesses should provide secure and attractive packaging (e.g., boxed or wrapped) of FVs because such products will more likely remain undamaged and encourage more customers to buy the product. 

For community markets, good zoning and organization of temporary open-air markets could effectively enhance their soundscape and acoustic comfort in high-density cities, which may promote the purchase of FVs in FV sales areas. Community markets should encourage the diversification of FVs, specifically those grown by farmers, who should adopt safety control measures that reduce health risks, including those stemming from the use of pesticides and heavy metals. They should also be willing to learning about consumer perceptions of FVs, including those concerning the effects of food safety risks and environmental impacts. For instance, an exhibition of local cultural features and freshness of locally grown FVs could increase consumer willingness to purchase FVs.

### 4.3. Government

Governments should institute policies, provide nutritional education, and create interventions that promote the availability of FVs, boost their consumption, and inform the public about healthy diets and food safety. They should encourage the establishment of sustainable food systems, effective food security, and urban nutrition policy that ensures greater accessibility to FVs. They should develop a database of consumer behaviours that mitigate health inequalities in which the health and consumption behaviours of social sub-groups may differ. They should target specific food environments such as ‘food deserts’, which lead to low FV consumption, particularly in socio-economically disadvantaged neighbourhoods. By implementing policies under the principle of ‘location proximity’ during urban planning, governments can establish food environments that provide customers with convenient yet healthy meals. In addition, to address consumers’ financial concerns, governments should attempt to control price fluctuations during the urbanization process, which could alter food prices. 

### 4.4. Community and Global Organizations

In addition to one-dimensional government policies, collaborations between community and global organizations can influence the dynamics of FV consumption. Cooperation between local and international certification and labelling authorities can lead to greater reliability of certification procedures and the traceability of FV products. Consistent labelling and certification of both domestic and exported FV products would ensure their credibility and deter forged or self-claimed labels, which could incur the distrust of consumers. Gaining the trust of consumers could entail the participation of NGOs and consumer protection agencies in both the horizontal and vertical coordination of standardized certification processes for FV products. Lastly, governments should establish national requirements for accurately labelling the calorie count and nutritional value by all food service businesses to ensure the reliability of FV dietary information. In general, the initiative to promote cooperation between the local community, NGOs, and research organizations should be strengthened.

## 5. Strengths and Limitations

To the best of our knowledge, this paper represents the first systematic review of study determinants associated with FV consumption in East and Southeast Asia in terms of facilitators, barriers, and moderators. One strength of the review is its focus on presenting a comprehensive view of FV consumption and food environments in an Asian setting; hence, we expect that our findings will foster constructive input for regional public health initiatives and government policymakers and support their efforts to formulate realistic policies that increase public FV consumption. As most of the studies we included in this review were published within the last five years, we consider the findings up to date; thus, they can be used to inform policymakers about timely policies. The review also provides a systematic classification of the determinants that influence FV consumption in food environments from the perspectives of individuals, food businesses, and the environments themselves. The numerous variables discussed in this review could represent concrete references that various sectors can consult to promote FV consumption. Nevertheless, this review was subject to several limitations. For one, even though we claimed that the countries in which the studies included in this review were conducted in shared similar dietary customs, the countries may have heterogeneous food environments and FV consumption habits. Nonetheless, aspiring developing countries can draw from the experiences and research conducted in more developed countries. As many of the studies included in the review took place in China, the variables that we identified might be representative of Chinese-specific lifestyles that other countries may not share.

## 6. Conclusions

This review aims to examine the determinants associated with FV consumption in East and Southeast Asian food environments by examining relevant facilitators, barriers, and moderators. The facilitators of FV consumption were the quality and safety of FVs and trust in their benefits perceived by consumers, as were food businesses with modernized food systems. Major moderators of individual FV consumption were demographics and shopping patterns; and major barriers to FV consumption were financial concerns stemming from high FV prices and urbanization-induced FV price inflation, the unavailability of food businesses selling FVs, and urbanized dietary patterns.

The proposed recommendations aim to increase FV consumption tailored to East and Southeast Asian contexts. Despite the seemingly segregated roles of different sectors, concerted and sustainable collaboration among the various stakeholders is the key to the successful implementation and sustenance of the above recommendations. Regional collaborations should be strengthened to build food environments favouring FV consumption.

Two research gaps were identified in this review. First, the findings of studies were often fragmented and conditionalized, hindering data integration, thus requiring standardized measurements of FV consumption for a sustainable and effective evaluation of FV consumption in East and Southeast Asian contexts. In addition, unlike Western countries, in which the impacts of FV consumption determinants and their interactions have been extensively studied, East and Southeast Asian countries have been substantially understudied. Therefore, this gap calls for more cross-sectional and longitudinal studies in countries, including China, and to a greater extent, Japan, Malaysia, South Korea, Taiwan, Thailand, and Vietnam. In other words, gaps in research on food environments and FV consumption in East and Southeast Asia demand scholarly attention.

## Figures and Tables

**Table 1:** The overall number of articles identified.

Database	13 May 2020	15 May 2020	Combined
Web of Science	1518	1598	3116
PubMed	694	542	1236
Scopus	2302	2533	4835
Cochrane Reviews	111	11	122
Cochrane Controlled Trials	172	38	210
CAB Abstracts	1344	1267	2611
Total	6141	5989	12,130
After removing duplicates			8469

**Table 2:** Quality Evaluation Protocol.

	Aspects	Choice of Answers	Scores Gained
QE1	The presence of evidence analyzed quantitatively or qualitatively	Quantitatively	2
		Qualitatively	1.5
		No Evidence	0
QE2	Reports of both added values and limitations of the study	Yes	2
		Partially (Only one side was reported)	1
		No	0
QE3	The justifiability of the outcomes (any involvement of techniques)	Yes	2
		Partially (Minimal explanation or details of techniques used)	1
		No	0
QE4	The reliability and recognizability of the publication source (Citation Number + H-Index)	>100	2
		50–99	1.5
		1–49	1
		0	0
QE5	Comparison of methodologies used in the study to other methodologies	Yes	1
		No	0

Remarks: QE = quality evaluation.

**Table 3:** Facilitators of FV consumption in East and Southeast Asia.

ID	Territory	Study Design	Sample Size	Individuals					Food Businesses						Environments		Reference
				(A) Perceived Quality, (B) Perceived Safety, (C) Good Value of Money, (D) Trust (Presence of Certificates), (E) High Socioeconomic Status of Consumers					(A) Online Platforms, (B) Daily Reliance, (C) FV Purchase Frequency at Wet Markets, (D) Convenience, (E) Food System Modernization, (F) Eating-out Culture						(A) Improved FV Accessibility, (B) Proximity to Markets/Supermarkets		
				(A)	(B)	(C)	(D)	(E)	(A)	(B)	(C)	(D)	(E)	(F)	(A)	(B)	
3	Malaysia	Cross-sectional	544	++	++	++											Chamhuri and Batt [28]
4	China	Cross-sectional	590				++										Cheng, Jiang [29]
6	Malaysia	Cross-sectional	700				++										Gindi, Abdullah [30]
8	China	Longitudinal	1680										++		++		Hovhannisyan and Devadoss [31]
10	Thailand	Longitudinal	1516								+						Kelly, Seubsman [32]
13	Taiwan	Cross-sectional	366						++								Lai, Pai [33]
14	Korea	Longitudinal	18,606											++			Lee and Kim [34]
17	China	Cross-sectional	212	++													Loebnitz and Grunert [35]
19	Japan	Cross-sectional	511 (control period); 704 (intervention period)			++											Nagatomo, Saito [36]
20	Japan	Longitudinal	Not Applicable										++				Ogawa, Tanabe [37]
22	China	Longitudinal	Not Applicable					++		++		++				++	Streeter [38]
23	Japan	Cross-sectional	24	++													Tait, Saunders [39]
24	Vietnam	Cross-sectional	300	++	++		++						++				Thai, Manh [40]
26	China	Cross-sectional	643						++								Wang and Somogyi [41]
27	Taiwan	Cross-sectional	800	++				++									Wann, Yang [42]
28	Vietnam	Cross-sectional	152 households; 24 consumers		++		++										Wertheim-Heck, Spaargaren [43]
29	Thailand	Cross-sectional	992	++			++										Wongprawmas and Canavari [44]
31	China	Cross-sectional	938				++										Yin, Hu [45]

Remarks: + = moderate association, ++ = strong association.

**Table 4:** Barriers to FV consumption in East and Southeast Asia.

ID	Territory	Study Design	Sample Size	Individuals			Food Businesses				Environments			Reference
				(A) Lack of Proficiency in Buying FVs, (B) Lack of Food Information, (C) Financial Concerns			(A) Perceived Complexity, (B) Subjective Loudness, (C) Lack of Validated Food Safety, (D) Unavailability of FVs				(A) Inflation due to Urbanization, (B) Urbanized Dietary Patterns, (C) Rurality			
				(A)	(B)	(C)	(A)	(B)	(C)	(D)	(A)	(B)	(C)	
1	Malaysia	Cross-sectional	1450								++			Azizan, Thangiah [46]
2	Vietnam	Longitudinal	25,899									++		Bairagi, Mohanty [47]
3	Malaysia	Cross-sectional	544			++								Chamhuri and Batt [28]
5	China	Longitudinal	>25,000									++		Dong and Fuller [48]
7	Vietnam	Cross-sectional	185	++										Hai, Moritaka [49]
8	China	Longitudinal	1680								++	++		Hovhannisyan and Devadoss [31]
9	China	Longitudinal	11,721										++	Huang and Tian [50]
11	Korea	Longitudinal	8616			++								Kim, Lee [51]
12	Korea	Cross-sectional	36 (lunchbox)							++				Kim and Choi [52]
13	Taiwan	Cross-sectional	366	++										Lai, Pai [33]
15	China	Cross-sectional	625 (food retailers)										++	Li, Song [53]
18	China	Cross-sectional	Not Applicable					++						Meng, Sun [54]
20	Japan	Longitudinal	Not Applicable							++				Ogawa, Tanabe [37]
22	China	Longitudinal	Not Applicable									++		Streeter [38]
24	Vietnam	Cross-sectional	300			++				++				Thai, Manh [40]
26	China	Cross-sectional	643				++							Wang and Somogyi [41]
28	Vietnam	Cross-sectional	152 households; 24 consumers		++				++					Wertheim-Heck, Spaargaren [43]
30	China	Longitudinal	216–217 (communities)								++			Wu, Xue [55]

Remarks: ++ = strong association.

**Table 5:** Moderators to FV consumption in East and Southeast Asia.

ID	Territory	Study Design	Sample Size	Individuals				Food Businesses				Environments	Reference
				(A) Lifestyle, (B) Demographics, (C) Subjective Norms, (D) Shopping Patterns				(A) Consumers’ Psychological Expectations, (B) Packaging, (C) Hygiene, (D) Relationship with Consumers				(A) Unique Geographic Landscape	
				(A)	(B)	(C)	(D)	(A)	(B)	(C)	(D)	(A)	
4	China	Cross-sectional	590		++		++						Cheng, Jiang [29]
6	Malaysia	Cross-sectional	700					++					Gindi, Abdullah [30]
7	Vietnam	Cross-sectional	185	++									Hai, Moritaka [49]
10	Thailand	Longitudinal	1516				++						Kelly, Seubsman [32]
11	Korea	Longitudinal	8616		++								Kim, Lee [51]
13	Taiwan	Cross-sectional	366		++								Lai, Pai [33]
14	Korea	Longitudinal	18,606		++								Lee and Kim [34]
17	China	Cross-sectional	212					++					Loebnitz and Grunert [35]
21	China	Cross-sectional	535			++							Qing, Lobo [56]
22	China	Longitudinal	Not Applicable	++					++	++			Streeter [38]
24	Vietnam	Cross-sectional	300								++		Thai, Manh [40]
25	China	Longitudinal	1007			++							Thogersen, Zhou [57]
26	China	Cross-sectional	643		++								Wang and Somogyi [41]
27	Taiwan	Cross-sectional	800									++	Wann, Yang [42]
28	Vietnam	Cross-sectional	152 households; 24 consumers				++				++		Wertheim-Heck, Spaargaren [43]

Remarks: ++ = strong association.

## Data Availability

Data sharing not applicable.

## Supplementary Materials

The following are available online at https://www.mdpi.com/2072-6643/13/1/148/s1, Table S1: Journals, Citations No., H-Index, and Quality Evaluation Scores.

## Appendix A

For the keywords ‘food environment’ and ‘fruit and vegetable’, this review included an exhaustive list of individual terms for comprehensiveness. The second search was an additional search with other five search terms about ‘food environment’ that were omitted in the first search: ‘marketing’, ‘market’, ‘school’, worksite’, and ‘workplace’.

*Database: Web of Science*
*Search date: 13 May, 2020*
*Search strategy:*

#1

TS=(“access to food” OR “accessibility of food” OR “availability of food” OR “burger joint*” OR cafe OR cafes OR cafeteria* OR canteen* OR carryout OR “catering enterprise*” OR “chain store*” OR “coffee house*” OR “coffee shop*” OR “convenience store*” OR “corner store*” OR “dessert house*” OR “dessert shop*” OR “eating environment*” OR “farmer market*” OR “farmer’s market*” OR “fast food*” OR “food access” OR “food availability” OR “food bank*” OR “food basket*” OR “food desert*” OR “food environment*” OR “food establishment*” OR “food facilit*” OR “food industr*” OR “food market*” OR “food operator*” OR “food outlet*” OR “food pantr*” OR “food retail*” OR “food service place*” OR “food service provid*” OR “food shop*” OR “food stall*” OR “food store*” OR “food swamp*” OR “food vend*” OR “food-rich environment*” OR “free market*” OR “fruit market*” OR “fruit store*” OR “greengrocer*” OR grocer* OR “icecream shop*” OR “icecream stand*” OR “icecream store*” OR “ice cream shop*” OR “ice cream stand*” OR “ice cream store*” OR “local food*” OR “market stall*” OR “nutrition environment*” OR “obesogenic environment*” OR “open market*” OR “open public market*” OR “open-air market*” OR pizzeria* OR restaurant* OR “retail food source*” OR “retail outlet*” OR “retailing environment*” OR “sandwich shop*” OR “shopping center*” OR “shopping centre*” OR “shopping mall*” OR “shopping street*” OR “snack bar” OR “snack bars” OR “snack shop*” OR “street vendor*” OR supermarket* OR “take out” OR “take away” OR “vegetable basket*” OR “vending machine*” OR “wet market*”)

100,049 results

#2

TS=(apple* OR “bamboo shoot*” OR banana* OR basil* OR bean* OR beet* OR “bell pepper*” OR berr* OR “black-eyed pea*” OR blackberr* OR “blood orange*” OR blueberr* OR boysenberr* OR breadfruit* OR broccoli* OR “brussels sprout*” OR cantaloupe* OR caper* OR carrot* OR cauliflower* OR celeriac* OR celery OR celeries OR chard* OR cherr* OR “chick pea*” OR “Chinese cabbage*” OR chive* OR citron* OR citrus OR coconut* OR “collard greens” OR crabapple* OR cranberr* OR cress* OR cucumber* OR currant* OR daikon* OR “dandelion green*” OR date OR dates OR “dragon fruit*” OR durian* OR “edible plant*” OR eggplant* OR elderberr* OR endive* OR “fava bean*” OR fennel* OR fig OR figs OR fruit* OR garlic* OR ginger* OR gourd* OR grape* OR “green bean*” OR greens OR guava* OR honeydew* OR “hot chili pepper*” OR “iceberg lettuce*” OR jackfruit* OR jicama* OR kiwi* OR kumquat* OR leek* OR legume* OR lemon* OR lentil* OR lettuce* OR “lima bean*” OR lime OR limes OR lingonberr* OR loquat* OR lychee* OR maize* OR “mandarin orange*” OR mango* OR marionberr* OR melon* OR mulberr* OR “mung bean*” OR mushroom* OR “mustard green*” OR nectarine* OR nut OR nuts OR okra* OR olive* OR onion* OR orange* OR papaya* OR parsley* OR parsnip* OR “passion fruit*” OR “pattypan squash*” OR pea OR peas OR peach* OR peanut* OR peapod* OR pear* OR pepper* OR persimmon* OR pickle* OR pineapple* OR plantain* OR plum* OR pluot* OR pomegranate* OR pomelo* OR potato* OR prune* OR pumpkin* OR quince* OR radicchio* OR radish* OR raisin* OR raspberr* OR rhubarb* OR rocket* OR romaine* OR rutabaga* OR salad* OR salsa* OR salsif* OR scallion* OR seaweed* OR shallot* OR sorrel* OR soybean* OR spinach* OR sprouts OR spuds OR squash OR squashes OR “star fruit*” OR strawberr* OR “string bean*” OR succotash* OR “sweet potato*” OR “Swiss chard*” OR tangelo* OR tangerine* OR taro* OR tomatillo* OR tomato* OR tuber OR tubers OR turnip* OR “ugli fruit*” OR vegetable* OR wasabi* OR “water chestnut*” OR watercress* OR watermelon* OR yam OR yams OR zucchini*)

2,491,581 results

#3

TS=(“South East Asia*” OR “Southeast Asia*” OR “Southeastern Asia*” OR Brunei* OR Cambodia* OR “East Timor” OR Indonesia* OR Laos OR Malaysia* OR Myanmar* OR Burma OR Burmese OR Philippine* OR Singapore* OR Thailand OR Thai OR Vietnam* OR “Viet Nam” OR Andaman* OR Nicobar* OR “Christmas Island” OR “Cocos Islands” OR “East Asia*” OR “Eastern Asia*” OR China OR Chinese OR “Hong Kong” OR Macau OR Mongolia* OR Korea* OR Japan* OR Taiwan*)

1,695,545 results

#4

#1 AND #2 AND #3

1560 results

#5

(#4) AND LANGUAGE: (English)

1518. results

*Database: Web of Science*
*Search date: 15 May, 2020*
*Search strategy:*

#1

TI=(market* OR school OR schools) OR TS=(worksite* OR “work site*” OR workplace* OR “work place*”)

446,410 results

#2

TS=(apple* OR “bamboo shoot*” OR banana* OR basil* OR bean* OR beet* OR “bell pepper*” OR berr* OR “black-eyed pea*” OR blackberr* OR “blood orange*” OR blueberr* OR boysenberr* OR breadfruit* OR broccoli* OR “brussels sprout*” OR cantaloupe* OR caper* OR carrot* OR cauliflower* OR celeriac* OR celery OR celeries OR chard* OR cherr* OR “chick pea*” OR “Chinese cabbage*” OR chive* OR citron* OR citrus OR coconut* OR “collard greens” OR crabapple* OR cranberr* OR cress* OR cucumber* OR currant* OR daikon* OR “dandelion green*” OR date OR dates OR “dragon fruit*” OR durian* OR “edible plant*” OR eggplant* OR elderberr* OR endive* OR “fava bean*” OR fennel* OR fig OR figs OR fruit* OR garlic* OR ginger* OR gourd* OR grape* OR “green bean*” OR greens OR guava* OR honeydew* OR “hot chili pepper*” OR “iceberg lettuce*” OR jackfruit* OR jicama* OR kiwi* OR kumquat* OR leek* OR legume* OR lemon* OR lentil* OR lettuce* OR “lima bean*” OR lime OR limes OR lingonberr* OR loquat* OR lychee* OR maize* OR “mandarin orange*” OR mango* OR marionberr* OR melon* OR mulberr* OR “mung bean*” OR mushroom* OR “mustard green*” OR nectarine* OR nut OR nuts OR okra* OR olive* OR onion* OR orange* OR papaya* OR parsley* OR parsnip* OR “passion fruit*” OR “pattypan squash*” OR pea OR peas OR peach* OR peanut* OR peapod* OR pear* OR pepper* OR persimmon* OR pickle* OR pineapple* OR plantain* OR plum* OR pluot* OR pomegranate* OR pomelo* OR potato* OR prune* OR pumpkin* OR quince* OR radicchio* OR radish* OR raisin* OR raspberr* OR rhubarb* OR rocket* OR romaine* OR rutabaga* OR salad* OR salsa* OR salsif* OR scallion* OR seaweed* OR shallot* OR sorrel* OR soybean* OR spinach* OR sprouts OR spuds OR squash OR squashes OR “star fruit*” OR strawberr* OR “string bean*” OR succotash* OR “sweet potato*” OR “Swiss chard*” OR tangelo* OR tangerine* OR taro* OR tomatillo* OR tomato* OR tuber OR tubers OR turnip* OR “ugli fruit*” OR vegetable* OR wasabi* OR “water chestnut*” OR watercress* OR watermelon* OR yam OR yams OR zucchini*)

2,492,940 results

#3

TS=(“South East Asia*” OR “Southeast Asia*” OR “Southeastern Asia*” OR Brunei* OR Cambodia* OR “East Timor” OR Indonesia* OR Laos OR Malaysia* OR Myanmar* OR Burma OR Burmese OR Philippine* OR Singapore* OR Thailand OR Thai OR Vietnam* OR “Viet Nam” OR Andaman* OR Nicobar* OR “Christmas Island” OR “Cocos Islands” OR “East Asia*” OR “Eastern Asia*” OR China OR Chinese OR “Hong Kong” OR Macau OR Mongolia* OR Korea* OR Japan* OR Taiwan*)

1,696,690 results

#4

#1 AND #2 AND #3

1649 results

#5

(#4) AND LANGUAGE: (English)

1598. results

*Database: PubMed*
*Search date: 13 May, 2020*
*Search strategy:*

#1

“access to food”[Text Word] OR “accessibility of food”[Text Word] OR “availability of food”[Text Word] OR “burger joint*”[Text Word] OR cafe[Text Word] OR cafes[Text Word] OR cafeteria*[Text Word] OR canteen*[Text Word] OR carryout[Text Word] OR “catering enterprise*”[Text Word] OR “chain store*”[Text Word] OR “coffee house*”[Text Word] OR “coffee shop*”[Text Word] OR “convenience store*”[Text Word] OR “corner store*”[Text Word] OR “dessert house*”[Text Word] OR “dessert shop*”[Text Word] OR “eating environment*”[Text Word] OR “farmer market*”[Text Word] OR “farmer’s market*”[Text Word] OR “fast food*”[Text Word] OR “food access”[Text Word] OR “food availability”[Text Word] OR “food bank*”[Text Word] OR “food basket*”[Text Word] OR “food desert*”[Text Word] OR “food environment*”[Text Word] OR “food establishment*”[Text Word] OR “food facilit*”[Text Word] OR “food industr*”[Text Word] OR “food market*”[Text Word] OR “food operator*”[Text Word] OR “food outlet*”[Text Word] OR “food pantr*”[Text Word] OR “food retail*”[Text Word] OR “food service place*”[Text Word] OR “food service provid*”[Text Word] OR “food shop*”[Text Word] OR “food stall*”[Text Word] OR “food store*”[Text Word] OR “food swamp*”[Text Word] OR “food vend*”[Text Word] OR “food-rich environment*”[Text Word] OR “free market*”[Text Word] OR “fruit market*”[Text Word] OR “fruit store*”[Text Word] OR “greengrocer*”[Text Word] OR grocer*[Text Word] OR “icecream shop*”[Text Word] OR “icecream stand*”[Text Word] OR “icecream store*”[Text Word] OR “ice cream shop*”[Text Word] OR “ice cream stand*”[Text Word] OR “ice cream store*”[Text Word] OR “local food*”[Text Word] OR “market stall*”[Text Word] OR “nutrition environment*”[Text Word] OR “obesogenic environment*”[Text Word] OR “open market*”[Text Word] OR “open public market*”[Text Word] OR “open-air market*”[Text Word] OR pizzeria*[Text Word] OR restaurant*[Text Word] OR “retail food source*”[Text Word] OR “retail outlet*”[Text Word] OR “retailing environment*”[Text Word] OR “sandwich shop*”[Text Word] OR “shopping center*”[Text Word] OR “shopping centre*”[Text Word] OR “shopping mall*”[Text Word] OR “shopping street*”[Text Word] OR “snack bar”[Text Word] OR “snack bars”[Text Word] OR “snack shop*”[Text Word] OR “street vendor*”[Text Word] OR supermarket*[Text Word] OR “take out”[Text Word] OR “take away”[Text Word] OR “vegetable basket*”[Text Word] OR “vending machine*”[Text Word] OR “wet market*”[Text Word]

48,304 results

#2

apple*[Text Word] OR “bamboo shoot*”[Text Word] OR banana*[Text Word] OR basil*[Text Word] OR bean*[Text Word] OR beet*[Text Word] OR “bell pepper*”[Text Word] OR berr* [Text Word] OR “black-eyed pea*”[Text Word] OR blackberr*[Text Word] OR “blood orange*”[Text Word] OR blueberr*[Text Word] OR boysenberr*[Text Word] OR breadfruit*[Text Word] OR broccoli*[Text Word] OR “brussels sprout*”[Text Word] OR cantaloupe*[Text Word] OR caper*[Text Word] OR carrot*[Text Word] OR cauliflower*[Text Word] OR celeriac*[Text Word] OR celery[Text Word] OR celeries[Text Word] OR chard*[Text Word] OR cherr*[Text Word] OR “chick pea*”[Text Word] OR “Chinese cabbage*”[Text Word] OR chive*[Text Word] OR citron*[Text Word] OR citrus[Text Word] OR coconut*[Text Word] OR “collard greens”[Text Word] OR crabapple*[Text Word] OR cranberr*[Text Word] OR cress*[Text Word] OR cucumber*[Text Word] OR currant*[Text Word] OR daikon*[Text Word] OR “dandelion green*”[Text Word] OR date[Text Word] OR dates[Text Word] OR “dragon fruit*”[Text Word] OR durian*[Text Word] OR “edible plant*”[Text Word] OR eggplant*[Text Word] OR elderberr*[Text Word] OR endive*[Text Word] OR “fava bean*”[Text Word] OR fennel*[Text Word] OR fig[Text Word] OR figs[Text Word] OR fruit*[Text Word] OR garlic*[Text Word] OR ginger*[Text Word] OR gourd*[Text Word] OR grape*[Text Word] OR “green bean*”[Text Word] OR greens[Text Word] OR guava*[Text Word] OR honeydew*[Text Word] OR “hot chili pepper*”[Text Word] OR “iceberg lettuce*”[Text Word] OR jackfruit*[Text Word] OR jicama*[Text Word] OR kiwi*[Text Word] OR kumquat*[Text Word] OR leek*[Text Word] OR legume*[Text Word] OR lemon*[Text Word] OR lentil*[Text Word] OR lettuce*[Text Word] OR “lima bean*”[Text Word] OR lime[Text Word] OR limes[Text Word] OR lingonberr*[Text Word] OR loquat*[Text Word] OR lychee*[Text Word] OR maize*[Text Word] OR “mandarin orange*”[Text Word] OR mango*[Text Word] OR marionberr*[Text Word] OR melon*[Text Word] OR mulberr*[Text Word] OR “mung bean*”[Text Word] OR mushroom*[Text Word] OR “mustard green*”[Text Word] OR nectarine*[Text Word] OR nut[Text Word] OR nuts[Text Word] OR okra*[Text Word] OR olive*[Text Word] OR onion*[Text Word] OR orange*[Text Word] OR papaya*[Text Word] OR parsley*[Text Word] OR parsnip*[Text Word] OR “passion fruit*”[Text Word] OR “pattypan squash*”[Text Word] OR pea[Text Word] OR peas[Text Word] OR peach*[Text Word] OR peanut*[Text Word] OR peapod*[Text Word] OR pear*[Text Word] OR pepper*[Text Word] OR persimmon*[Text Word] OR pickle*[Text Word] OR pineapple*[Text Word] OR plantain*[Text Word] OR plum*[Text Word] OR pluot*[Text Word] OR pomegranate*[Text Word] OR pomelo*[Text Word] OR potato*[Text Word] OR prune*[Text Word] OR pumpkin*[Text Word] OR quince*[Text Word] OR radicchio*[Text Word] OR radish*[Text Word] OR raisin*[Text Word] OR raspberr*[Text Word] OR rhubarb*[Text Word] OR rocket*[Text Word] OR romaine*[Text Word] OR rutabaga*[Text Word] OR salad*[Text Word] OR salsa*[Text Word] OR salsif*[Text Word] OR scallion*[Text Word] OR seaweed*[Text Word] OR shallot*[Text Word] OR sorrel*[Text Word] OR soybean*[Text Word] OR spinach*[Text Word] OR sprouts[Text Word] OR spuds[Text Word] OR squash[Text Word] OR squashes[Text Word] OR “star fruit*”[Text Word] OR strawberr*[Text Word] OR “string bean*”[Text Word] OR succotash*[Text Word] OR “sweet potato*”[Text Word] OR “Swiss chard*”[Text Word] OR tangelo*[Text Word] OR tangerine*[Text Word] OR taro*[Text Word] OR tomatillo*[Text Word] OR tomato*[Text Word] OR tuber[Text Word] OR tubers[Text Word] OR turnip*[Text Word] OR “ugli fruit*”[Text Word] OR vegetable*[Text Word] OR wasabi*[Text Word] OR “water chestnut*”[Text Word] OR watercress*[Text Word] OR watermelon*[Text Word] OR yam[Text Word] OR yams[Text Word] OR zucchini*[Text Word]

962,570 results

#3

“South East Asia*”[Text Word] OR “Southeast Asia*”[Text Word] OR “Southeastern Asia*”[Text Word] OR Brunei*[Text Word] OR Cambodia*[Text Word] OR “East Timor”[Text Word] OR Indonesia*[Text Word] OR Laos[Text Word] OR Malaysia*[Text Word] OR Myanmar*[Text Word] OR Burma[Text Word] OR Burmese[Text Word] OR Philippine*[Text Word] OR Singapore*[Text Word] OR Thailand[Text Word] OR Thai[Text Word] OR Vietnam*[Text Word] OR “Viet Nam”[Text Word] OR Andaman*[Text Word] OR Nicobar*[Text Word] OR “Christmas Island”[Text Word] OR “Cocos Islands”[Text Word] OR “East Asia*”[Text Word] OR “Eastern Asia*”[Text Word] OR China[Text Word] OR Chinese[Text Word] OR “Hong Kong”[Text Word] OR Macau[Text Word] OR Mongolia*[Text Word] OR Korea*[Text Word] OR Japan*[Text Word] OR Taiwan*[Text Word]

923,507 results

#4

#1 AND #2 AND #3

732 results

#5

#4 AND English[Language]

694. results

*Database: PubMed*
*Search date: 15 May, 2020*
*Search strategy:*

#1

market*[Title] OR school[Title] OR schools[Title] OR worksite*[Text Word] OR “work site*”[Text Word] OR workplace*[Text Word] OR “work place*”[Text Word]

178,040 results

#2

apple*[Text Word] OR “bamboo shoot*”[Text Word] OR banana*[Text Word] OR basil*[Text Word] OR bean*[Text Word] OR beet*[Text Word] OR “bell pepper*”[Text Word] OR berr* [Text Word] OR “black-eyed pea*”[Text Word] OR blackberr*[Text Word] OR “blood orange*”[Text Word] OR blueberr*[Text Word] OR boysenberr*[Text Word] OR breadfruit*[Text Word] OR broccoli*[Text Word] OR “brussels sprout*”[Text Word] OR cantaloupe*[Text Word] OR caper*[Text Word] OR carrot*[Text Word] OR cauliflower*[Text Word] OR celeriac*[Text Word] OR celery[Text Word] OR celeries[Text Word] OR chard*[Text Word] OR cherr*[Text Word] OR “chick pea*”[Text Word] OR “Chinese cabbage*”[Text Word] OR chive*[Text Word] OR citron*[Text Word] OR citrus[Text Word] OR coconut*[Text Word] OR “collard greens”[Text Word] OR crabapple*[Text Word] OR cranberr*[Text Word] OR cress*[Text Word] OR cucumber*[Text Word] OR currant*[Text Word] OR daikon*[Text Word] OR “dandelion green*”[Text Word] OR date[Text Word] OR dates[Text Word] OR “dragon fruit*”[Text Word] OR durian*[Text Word] OR “edible plant*”[Text Word] OR eggplant*[Text Word] OR elderberr*[Text Word] OR endive*[Text Word] OR “fava bean*”[Text Word] OR fennel*[Text Word] OR fig[Text Word] OR figs[Text Word] OR fruit*[Text Word] OR garlic*[Text Word] OR ginger*[Text Word] OR gourd*[Text Word] OR grape*[Text Word] OR “green bean*”[Text Word] OR greens[Text Word] OR guava*[Text Word] OR honeydew*[Text Word] OR “hot chili pepper*”[Text Word] OR “iceberg lettuce*”[Text Word] OR jackfruit*[Text Word] OR jicama*[Text Word] OR kiwi*[Text Word] OR kumquat*[Text Word] OR leek*[Text Word] OR legume*[Text Word] OR lemon*[Text Word] OR lentil*[Text Word] OR lettuce*[Text Word] OR “lima bean*”[Text Word] OR lime[Text Word] OR limes[Text Word] OR lingonberr*[Text Word] OR loquat*[Text Word] OR lychee*[Text Word] OR maize*[Text Word] OR “mandarin orange*”[Text Word] OR mango*[Text Word] OR marionberr*[Text Word] OR melon*[Text Word] OR mulberr*[Text Word] OR “mung bean*”[Text Word] OR mushroom*[Text Word] OR “mustard green*”[Text Word] OR nectarine*[Text Word] OR nut[Text Word] OR nuts[Text Word] OR okra*[Text Word] OR olive*[Text Word] OR onion*[Text Word] OR orange*[Text Word] OR papaya*[Text Word] OR parsley*[Text Word] OR parsnip*[Text Word] OR “passion fruit*”[Text Word] OR “pattypan squash*”[Text Word] OR pea[Text Word] OR peas[Text Word] OR peach*[Text Word] OR peanut*[Text Word] OR peapod*[Text Word] OR pear*[Text Word] OR pepper*[Text Word] OR persimmon*[Text Word] OR pickle*[Text Word] OR pineapple*[Text Word] OR plantain*[Text Word] OR plum*[Text Word] OR pluot*[Text Word] OR pomegranate*[Text Word] OR pomelo*[Text Word] OR potato*[Text Word] OR prune*[Text Word] OR pumpkin*[Text Word] OR quince*[Text Word] OR radicchio*[Text Word] OR radish*[Text Word] OR raisin*[Text Word] OR raspberr*[Text Word] OR rhubarb*[Text Word] OR rocket*[Text Word] OR romaine*[Text Word] OR rutabaga*[Text Word] OR salad*[Text Word] OR salsa*[Text Word] OR salsif*[Text Word] OR scallion*[Text Word] OR seaweed*[Text Word] OR shallot*[Text Word] OR sorrel*[Text Word] OR soybean*[Text Word] OR spinach*[Text Word] OR sprouts[Text Word] OR spuds[Text Word] OR squash[Text Word] OR squashes[Text Word] OR “star fruit*”[Text Word] OR strawberr*[Text Word] OR “string bean*”[Text Word] OR succotash*[Text Word] OR “sweet potato*”[Text Word] OR “Swiss chard*”[Text Word] OR tangelo*[Text Word] OR tangerine*[Text Word] OR taro*[Text Word] OR tomatillo*[Text Word] OR tomato*[Text Word] OR tuber[Text Word] OR tubers[Text Word] OR turnip*[Text Word] OR “ugli fruit*”[Text Word] OR vegetable*[Text Word] OR wasabi*[Text Word] OR “water chestnut*”[Text Word] OR watercress*[Text Word] OR watermelon*[Text Word] OR yam[Text Word] OR yams[Text Word] OR zucchini*[Text Word]

963,311 results

#3

“South East Asia*”[Text Word] OR “Southeast Asia*”[Text Word] OR “Southeastern Asia*”[Text Word] OR Brunei*[Text Word] OR Cambodia*[Text Word] OR “East Timor”[Text Word] OR Indonesia*[Text Word] OR Laos[Text Word] OR Malaysia*[Text Word] OR Myanmar*[Text Word] OR Burma[Text Word] OR Burmese[Text Word] OR Philippine*[Text Word] OR Singapore*[Text Word] OR Thailand[Text Word] OR Thai[Text Word] OR Vietnam*[Text Word] OR “Viet Nam”[Text Word] OR Andaman*[Text Word] OR Nicobar*[Text Word] OR “Christmas Island”[Text Word] OR “Cocos Islands”[Text Word] OR “East Asia*”[Text Word] OR “Eastern Asia*”[Text Word] OR China[Text Word] OR Chinese[Text Word] OR “Hong Kong”[Text Word] OR Macau[Text Word] OR Mongolia*[Text Word] OR Korea*[Text Word] OR Japan*[Text Word] OR Taiwan*[Text Word]

924,400 results

#4

#1 AND #2 AND #3

605 results

#5

#4 AND English[Language]

542. results

*Database: Scopus*
*Search date: 13 May, 2020*
*Search strategy:*

#1

TITLE-ABS(“access to food” OR “accessibility of food” OR “availability of food” OR “burger joint*” OR cafe OR cafes OR cafeteria* OR canteen* OR carryout OR “catering enterprise*” OR “chain store*” OR “coffee house*” OR “coffee shop*” OR “convenience store*” OR “corner store*” OR “dessert house*” OR “dessert shop*” OR “eating environment*” OR “farmer market*” OR “farmer’s market*” OR “fast food*” OR “food access” OR “food availability” OR “food bank*” OR “food basket*” OR “food desert*” OR “food environment*” OR “food establishment*” OR “food facilit*” OR “food industr*” OR “food market*” OR “food operator*” OR “food outlet*” OR “food pantr*” OR “food retail*” OR “food service place*” OR “food service provid*” OR “food shop*” OR “food stall*” OR “food store*” OR “food swamp*” OR “food vend*” OR “food-rich environment*” OR “free market*” OR “fruit market*” OR “fruit store*” OR “greengrocer*” OR grocer* OR “icecream shop*” OR “icecream stand*” OR “icecream store*” OR “ice cream shop*” OR “ice cream stand*” OR “ice cream store*” OR “local food*” OR “market stall*” OR “nutrition environment*” OR “obesogenic environment*” OR “open market*” OR “open public market*” OR “open-air market*” OR pizzeria* OR restaurant* OR “retail food source*” OR “retail outlet*” OR “retailing environment*” OR “sandwich shop*” OR “shopping center*” OR “shopping centre*” OR “shopping mall*” OR “shopping street*” OR “snack bar” OR “snack bars” OR “snack shop*” OR “street vendor*” OR supermarket* OR “take out” OR “take away” OR “vegetable basket*” OR “vending machine*” OR “wet market*”)

153,253 results

#2

TITLE-ABS(apple* OR “bamboo shoot*” OR banana* OR basil* OR bean* OR beet* OR “bell pepper*” OR berr* OR “black-eyed pea*” OR blackberr* OR “blood orange*” OR blueberr* OR boysenberr* OR breadfruit* OR broccoli* OR “brussels sprout*” OR cantaloupe* OR caper* OR carrot* OR cauliflower* OR celeriac* OR celery OR celeries OR chard* OR cherr* OR “chick pea*” OR “Chinese cabbage*” OR chive* OR citron* OR citrus OR coconut* OR “collard greens” OR crabapple* OR cranberr* OR cress* OR cucumber* OR currant* OR daikon* OR “dandelion green*” OR date OR dates OR “dragon fruit*” OR durian* OR “edible plant*” OR eggplant* OR elderberr* OR endive* OR “fava bean*” OR fennel* OR fig OR figs OR fruit* OR garlic* OR ginger* OR gourd* OR grape* OR “green bean*” OR greens OR guava* OR honeydew* OR “hot chili pepper*” OR “iceberg lettuce*” OR jackfruit* OR jicama* OR kiwi* OR kumquat* OR leek* OR legume* OR lemon* OR lentil* OR lettuce* OR “lima bean*” OR lime OR limes OR lingonberr* OR loquat* OR lychee* OR maize* OR “mandarin orange*” OR mango* OR marionberr* OR melon* OR mulberr* OR “mung bean*” OR mushroom* OR “mustard green*” OR nectarine* OR nut OR nuts OR okra* OR olive* OR onion* OR orange* OR papaya* OR parsley* OR parsnip* OR “passion fruit*” OR “pattypan squash*” OR pea OR peas OR peach* OR peanut* OR peapod* OR pear* OR pepper* OR persimmon* OR pickle* OR pineapple* OR plantain* OR plum* OR pluot* OR pomegranate* OR pomelo* OR potato* OR prune* OR pumpkin* OR quince* OR radicchio* OR radish* OR raisin* OR raspberr* OR rhubarb* OR rocket* OR romaine* OR rutabaga* OR salad* OR salsa* OR salsif* OR scallion* OR seaweed* OR shallot* OR sorrel* OR soybean* OR spinach* OR sprouts OR spuds OR squash OR squashes OR “star fruit*” OR strawberr* OR “string bean*” OR succotash* OR “sweet potato*” OR “Swiss chard*” OR tangelo* OR tangerine* OR taro* OR tomatillo* OR tomato* OR tuber OR tubers OR turnip* OR “ugli fruit*” OR vegetable* OR wasabi* OR “water chestnut*” OR watercress* OR watermelon* OR yam OR yams OR zucchini*)

3,074,443 results

#3

TITLE-ABS(“South East Asia*” OR “Southeast Asia*” OR “Southeastern Asia*” OR Brunei* OR Cambodia* OR “East Timor” OR Indonesia* OR Laos OR Malaysia* OR Myanmar* OR Burma OR Burmese OR Philippine* OR Singapore* OR Thailand OR Thai OR Vietnam* OR “Viet Nam” OR Andaman* OR Nicobar* OR “Christmas Island” OR “Cocos Islands” OR “East Asia*” OR “Eastern Asia*” OR China OR Chinese OR “Hong Kong” OR Macau OR Mongolia* OR Korea* OR Japan* OR Taiwan*)

3,330,231 results

#4

#1 AND #2 AND #3

2514 results

#5

LIMIT #4 TO LANGUAGE(English)

2302. Results

*Database: Scopus*
*Search date: 15 May, 2020*
*Search strategy:*

#1

TITLE(market* OR school OR schools) OR TITLE-ABS(worksite* OR “work site*” OR workplace* OR “work place*”)

643,809 results

#2

TITLE-ABS(apple* OR “bamboo shoot*” OR banana* OR basil* OR bean* OR beet* OR “bell pepper*” OR berr* OR “black-eyed pea*” OR blackberr* OR “blood orange*” OR blueberr* OR boysenberr* OR breadfruit* OR broccoli* OR “brussels sprout*” OR cantaloupe* OR caper* OR carrot* OR cauliflower* OR celeriac* OR celery OR celeries OR chard* OR cherr* OR “chick pea*” OR “Chinese cabbage*” OR chive* OR citron* OR citrus OR coconut* OR “collard greens” OR crabapple* OR cranberr* OR cress* OR cucumber* OR currant* OR daikon* OR “dandelion green*” OR date OR dates OR “dragon fruit*” OR durian* OR “edible plant*” OR eggplant* OR elderberr* OR endive* OR “fava bean*” OR fennel* OR fig OR figs OR fruit* OR garlic* OR ginger* OR gourd* OR grape* OR “green bean*” OR greens OR guava* OR honeydew* OR “hot chili pepper*” OR “iceberg lettuce*” OR jackfruit* OR jicama* OR kiwi* OR kumquat* OR leek* OR legume* OR lemon* OR lentil* OR lettuce* OR “lima bean*” OR lime OR limes OR lingonberr* OR loquat* OR lychee* OR maize* OR “mandarin orange*” OR mango* OR marionberr* OR melon* OR mulberr* OR “mung bean*” OR mushroom* OR “mustard green*” OR nectarine* OR nut OR nuts OR okra* OR olive* OR onion* OR orange* OR papaya* OR parsley* OR parsnip* OR “passion fruit*” OR “pattypan squash*” OR pea OR peas OR peach* OR peanut* OR peapod* OR pear* OR pepper* OR persimmon* OR pickle* OR pineapple* OR plantain* OR plum* OR pluot* OR pomegranate* OR pomelo* OR potato* OR prune* OR pumpkin* OR quince* OR radicchio* OR radish* OR raisin* OR raspberr* OR rhubarb* OR rocket* OR romaine* OR rutabaga* OR salad* OR salsa* OR salsif* OR scallion* OR seaweed* OR shallot* OR sorrel* OR soybean* OR spinach* OR sprouts OR spuds OR squash OR squashes OR “star fruit*” OR strawberr* OR “string bean*” OR succotash* OR “sweet potato*” OR “Swiss chard*” OR tangelo* OR tangerine* OR taro* OR tomatillo* OR tomato* OR tuber OR tubers OR turnip* OR “ugli fruit*” OR vegetable* OR wasabi* OR “water chestnut*” OR watercress* OR watermelon* OR yam OR yams OR zucchini*)

3,075,704 results

#3

TITLE-ABS(“South East Asia*” OR “Southeast Asia*” OR “Southeastern Asia*” OR Brunei* OR Cambodia* OR “East Timor” OR Indonesia* OR Laos OR Malaysia* OR Myanmar* OR Burma OR Burmese OR Philippine* OR Singapore* OR Thailand OR Thai OR Vietnam* OR “Viet Nam” OR Andaman* OR Nicobar* OR “Christmas Island” OR “Cocos Islands” OR “East Asia*” OR “Eastern Asia*” OR China OR Chinese OR “Hong Kong” OR Macau OR Mongolia* OR Korea* OR Japan* OR Taiwan*)

3,331,600 results

#4

#1 AND #2 AND #3

2732 results

#5

LIMIT #4 TO LANGUAGE(English)

2533. Results

*Databases:*
  (1) *Cochrane Database of Systematic Reviews, Issue 5 of 12, May 2020*
  (2) *Cochrane Central Register of Controlled Trials, Issue 5 of 12, May 2020*
*Search date: 13 May, 2020*
*Search strategy:*

#1

“access to food” OR “accessibility of food” OR “availability of food” OR “burger joint*” OR cafe OR cafes OR cafeteria* OR canteen* OR carryout OR “catering enterprise*” OR “chain store*” OR “coffee house*” OR “coffee shop*” OR “convenience store*” OR “corner store*” OR “dessert house*” OR “dessert shop*” OR “eating environment*” OR “farmer market*” OR “farmer’s market*” OR “fast food*” OR “food access” OR “food availability” OR “food bank*” OR “food basket*” OR “food desert*” OR “food environment*” OR “food establishment*” OR “food facilit*” OR “food industr*” OR “food market*” OR “food operator*” OR “food outlet*” OR “food pantr*” OR “food retail*” OR “food service place*” OR “food service provid*” OR “food shop*” OR “food stall*” OR “food store*” OR “food swamp*” OR “food vend*” OR “food-rich environment*” OR “free market*” OR “fruit market*” OR “fruit store*” OR “greengrocer*” OR grocer* OR “icecream shop*” OR “icecream stand*” OR “icecream store*” OR “ice cream shop*” OR “ice cream stand*” OR “ice cream store*” OR “local food*” OR “market stall*” OR “nutrition environment*” OR “obesogenic environment*” OR “open market*” OR “open public market*” OR “open-air market*” OR pizzeria* OR restaurant* OR “retail food source*” OR “retail outlet*” OR “retailing environment*” OR “sandwich shop*” OR “shopping center*” OR “shopping centre*” OR “shopping mall*” OR “shopping street*” OR “snack bar” OR “snack bars” OR “snack shop*” OR “street vendor*” OR supermarket* OR “take out” OR “take away” OR “vegetable basket*” OR “vending machine*” OR “wet market*”

2512 results

#2

apple* OR “bamboo shoot*” OR banana* OR basil* OR bean* OR beet* OR “bell pepper*” OR berr* OR “black-eyed pea*” OR blackberr* OR “blood orange*” OR blueberr* OR boysenberr* OR breadfruit* OR broccoli* OR “brussels sprout*” OR cantaloupe* OR caper* OR carrot* OR cauliflower* OR celeriac* OR celery OR celeries OR chard* OR cherr* OR “chick pea*” OR “Chinese cabbage*” OR chive* OR citron* OR citrus OR coconut* OR “collard greens” OR crabapple* OR cranberr* OR cress* OR cucumber* OR currant* OR daikon* OR “dandelion green*” OR date OR dates OR “dragon fruit*” OR durian* OR “edible plant*” OR eggplant* OR elderberr* OR endive* OR “fava bean*” OR fennel* OR fig OR figs OR fruit* OR garlic* OR ginger* OR gourd* OR grape* OR “green bean*” OR greens OR guava* OR honeydew* OR “hot chili pepper*” OR “iceberg lettuce*” OR jackfruit* OR jicama* OR kiwi* OR kumquat* OR leek* OR legume* OR lemon* OR lentil* OR lettuce* OR “lima bean*” OR lime OR limes OR lingonberr* OR loquat* OR lychee* OR maize* OR “mandarin orange*” OR mango* OR marionberr* OR melon* OR mulberr* OR “mung bean*” OR mushroom* OR “mustard green*” OR nectarine* OR nut OR nuts OR okra* OR olive* OR onion* OR orange* OR papaya* OR parsley* OR parsnip* OR “passion fruit*” OR “pattypan squash*” OR pea OR peas OR peach* OR peanut* OR peapod* OR pear* OR pepper* OR persimmon* OR pickle* OR pineapple* OR plantain* OR plum* OR pluot* OR pomegranate* OR pomelo* OR potato* OR prune* OR pumpkin* OR quince* OR radicchio* OR radish* OR raisin* OR raspberr* OR rhubarb* OR rocket* OR romaine* OR rutabaga* OR salad* OR salsa* OR salsif* OR scallion* OR seaweed* OR shallot* OR sorrel* OR soybean* OR spinach* OR sprouts OR spuds OR squash OR squashes OR “star fruit*” OR strawberr* OR “string bean*” OR succotash* OR “sweet potato*” OR “Swiss chard*” OR tangelo* OR tangerine* OR taro* OR tomatillo* OR tomato* OR tuber OR tubers OR turnip* OR “ugli fruit*” OR vegetable* OR wasabi* OR “water chestnut*” OR watercress* OR watermelon* OR yam OR yams OR zucchini*

1,632,996 results

#3

“South East Asia*” OR “Southeast Asia*” OR “Southeastern Asia*” OR Brunei* OR Cambodia* OR “East Timor” OR Indonesia* OR Laos OR Malaysia* OR Myanmar* OR Burma OR Burmese OR Philippine* OR Singapore* OR Thailand OR Thai OR Vietnam* OR “Viet Nam” OR Andaman* OR Nicobar* OR “Christmas Island” OR “Cocos Islands” OR “East Asia*” OR “Eastern Asia*” OR China OR Chinese OR “Hong Kong” OR Macau OR Mongolia* OR Korea* OR Japan* OR Taiwan*

166,889 results

#4

#1 AND #2 AND #3

111 Cochrane reviews

172. Controlled trials

*Databases:*
  (1) *Cochrane Database of Systematic Reviews, Issue 5 of 12, May 2020*
  (2) *Cochrane Central Register of Controlled Trials, Issue 5 of 12, May 2020*
*Search date: 15 May, 2020*
*Search strategy:*

#1

(market* OR school OR schools):ti OR (worksite* OR “work site*” OR workplace* OR “work place*”):ti,ab,kw

11,758 results

#2

(apple* OR “bamboo shoot*” OR banana* OR basil* OR bean* OR beet* OR “bell pepper*” OR berr* OR “black-eyed pea*” OR blackberr* OR “blood orange*” OR blueberr* OR boysenberr* OR breadfruit* OR broccoli* OR “brussels sprout*” OR cantaloupe* OR caper* OR carrot* OR cauliflower* OR celeriac* OR celery OR celeries OR chard* OR cherr* OR “chick pea*” OR “Chinese cabbage*” OR chive* OR citron* OR citrus OR coconut* OR “collard greens” OR crabapple* OR cranberr* OR cress* OR cucumber* OR currant* OR daikon* OR “dandelion green*” OR date OR dates OR “dragon fruit*” OR durian* OR “edible plant*” OR eggplant* OR elderberr* OR endive* OR “fava bean*” OR fennel* OR fig OR figs OR fruit* OR garlic* OR ginger* OR gourd* OR grape* OR “green bean*” OR greens OR guava* OR honeydew* OR “hot chili pepper*” OR “iceberg lettuce*” OR jackfruit* OR jicama* OR kiwi* OR kumquat* OR leek* OR legume* OR lemon* OR lentil* OR lettuce* OR “lima bean*” OR lime OR limes OR lingonberr* OR loquat* OR lychee* OR maize* OR “mandarin orange*” OR mango* OR marionberr* OR melon* OR mulberr* OR “mung bean*” OR mushroom* OR “mustard green*” OR nectarine* OR nut OR nuts OR okra* OR olive* OR onion* OR orange* OR papaya* OR parsley* OR parsnip* OR “passion fruit*” OR “pattypan squash*” OR pea OR peas OR peach* OR peanut* OR peapod* OR pear* OR pepper* OR persimmon* OR pickle* OR pineapple* OR plantain* OR plum* OR pluot* OR pomegranate* OR pomelo* OR potato* OR prune* OR pumpkin* OR quince* OR radicchio* OR radish* OR raisin* OR raspberr* OR rhubarb* OR rocket* OR romaine* OR rutabaga* OR salad* OR salsa* OR salsif* OR scallion* OR seaweed* OR shallot* OR sorrel* OR soybean* OR spinach* OR sprouts OR spuds OR squash OR squashes OR “star fruit*” OR strawberr* OR “string bean*” OR succotash* OR “sweet potato*” OR “Swiss chard*” OR tangelo* OR tangerine* OR taro* OR tomatillo* OR tomato* OR tuber OR tubers OR turnip* OR “ugli fruit*” OR vegetable* OR wasabi* OR “water chestnut*” OR watercress* OR watermelon* OR yam OR yams OR zucchini*):ti,ab,kw

70,693 results

#3

(“South East Asia*” OR “Southeast Asia*” OR “Southeastern Asia*” OR Brunei* OR Cambodia* OR “East Timor” OR Indonesia* OR Laos OR Malaysia* OR Myanmar* OR Burma OR Burmese OR Philippine* OR Singapore* OR Thailand OR Thai OR Vietnam* OR “Viet Nam” OR Andaman* OR Nicobar* OR “Christmas Island” OR “Cocos Islands” OR “East Asia*” OR “Eastern Asia*” OR China OR Chinese OR “Hong Kong” OR Macau OR Mongolia* OR Korea* OR Japan* OR Taiwan*):ti,ab,kw

72,475 results

#4

#1 AND #2 AND #3

11 Cochrane reviews

38. Controlled trials

*Database: CAB Abstracts 1973 to 2020 Week 18*
*Search date: 13 May, 2020*
*Search strategy:*

1

(“access to food” OR “accessibility of food” OR “availability of food” OR “burger joint*” OR cafe OR cafes OR cafeteria* OR canteen* OR carryout OR “catering enterprise*” OR “chain store*” OR “coffee house*” OR “coffee shop*” OR “convenience store*” OR “corner store*” OR “dessert house*” OR “dessert shop*” OR “eating environment*” OR “farmer market*” OR “farmer’s market*” OR “fast food*” OR “food access” OR “food availability” OR “food bank*” OR “food basket*” OR “food desert*” OR “food environment*” OR “food establishment*” OR “food facilit*” OR “food industr*” OR “food market*” OR “food operator*” OR “food outlet*” OR “food pantr*” OR “food retail*” OR “food service place*” OR “food service provid*” OR “food shop*” OR “food stall*” OR “food store*” OR “food swamp*” OR “food vend*” OR “food-rich environment*” OR “free market*” OR “fruit market*” OR “fruit store*” OR “greengrocer*” OR grocer* OR “icecream shop*” OR “icecream stand*” OR “icecream store*” OR “ice cream shop*” OR “ice cream stand*” OR “ice cream store*” OR “local food*” OR “market stall*” OR “nutrition environment*” OR “obesogenic environment*” OR “open market*” OR “open public market*” OR “open-air market*” OR pizzeria* OR restaurant* OR “retail food source*” OR “retail outlet*” OR “retailing environment*” OR “sandwich shop*” OR “shopping center*” OR “shopping centre*” OR “shopping mall*” OR “shopping street*” OR “snack bar” OR “snack bars” OR “snack shop*” OR “street vendor*” OR supermarket* OR “take out” OR “take away” OR “vegetable basket*” OR “vending machine*” OR “wet market*”).ti,ab.

80,418 results

2

(apple* OR “bamboo shoot*” OR banana* OR basil* OR bean* OR beet* OR “bell pepper*” OR berr* OR “black-eyed pea*” OR blackberr* OR “blood orange*” OR blueberr* OR boysenberr* OR breadfruit* OR broccoli* OR “brussels sprout*” OR cantaloupe* OR caper* OR carrot* OR cauliflower* OR celeriac* OR celery OR celeries OR chard* OR cherr* OR “chick pea*” OR “Chinese cabbage*” OR chive* OR citron* OR citrus OR coconut* OR “collard greens” OR crabapple* OR cranberr* OR cress* OR cucumber* OR currant* OR daikon* OR “dandelion green*” OR date OR dates OR “dragon fruit*” OR durian* OR “edible plant*” OR eggplant* OR elderberr* OR endive* OR “fava bean*” OR fennel* OR fig OR figs OR fruit* OR garlic* OR ginger* OR gourd* OR grape* OR “green bean*” OR greens OR guava* OR honeydew* OR “hot chili pepper*” OR “iceberg lettuce*” OR jackfruit* OR jicama* OR kiwi* OR kumquat* OR leek* OR legume* OR lemon* OR lentil* OR lettuce* OR “lima bean*” OR lime OR limes OR lingonberr* OR loquat* OR lychee* OR maize* OR “mandarin orange*” OR mango* OR marionberr* OR melon* OR mulberr* OR “mung bean*” OR mushroom* OR “mustard green*” OR nectarine* OR nut OR nuts OR okra* OR olive* OR onion* OR orange* OR papaya* OR parsley* OR parsnip* OR “passion fruit*” OR “pattypan squash*” OR pea OR peas OR peach* OR peanut* OR peapod* OR pear* OR pepper* OR persimmon* OR pickle* OR pineapple* OR plantain* OR plum* OR pluot* OR pomegranate* OR pomelo* OR potato* OR prune* OR pumpkin* OR quince* OR radicchio* OR radish* OR raisin* OR raspberr* OR rhubarb* OR rocket* OR romaine* OR rutabaga* OR salad* OR salsa* OR salsif* OR scallion* OR seaweed* OR shallot* OR sorrel* OR soybean* OR spinach* OR sprouts OR spuds OR squash OR squashes OR “star fruit*” OR strawberr* OR “string bean*” OR succotash* OR “sweet potato*” OR “Swiss chard*” OR tangelo* OR tangerine* OR taro* OR tomatillo* OR tomato* OR tuber OR tubers OR turnip* OR “ugli fruit*” OR vegetable* OR wasabi* OR “water chestnut*” OR watercress* OR watermelon* OR yam OR yams OR zucchini*).ti,ab.

1,878,056 results

3

(“South East Asia*” OR “Southeast Asia*” OR “Southeastern Asia*” OR Brunei* OR Cambodia* OR “East Timor” OR Indonesia* OR Laos OR Malaysia* OR Myanmar* OR Burma OR Burmese OR Philippine* OR Singapore* OR Thailand OR Thai OR Vietnam* OR “Viet Nam” OR Andaman* OR Nicobar* OR “Christmas Island” OR “Cocos Islands” OR “East Asia*” OR “Eastern Asia*” OR China OR Chinese OR “Hong Kong” OR Macau OR Mongolia* OR Korea* OR Japan* OR Taiwan*).ti,ab.

651,739 results

4

1 AND 2 AND 3

1622 results

5 limit 4 to English language

1344. Results

*Database: CAB Abstracts 1973 to 2020 Week 18*
*Search date: 15 May, 2020*
*Search strategy:*

1

(market* OR school OR schools).ti. OR (worksite* OR “work site*” OR workplace* OR “work place*”).ti,ab.

90,400 results

2

(apple* OR “bamboo shoot*” OR banana* OR basil* OR bean* OR beet* OR “bell pepper*” OR berr* OR “black-eyed pea*” OR blackberr* OR “blood orange*” OR blueberr* OR boysenberr* OR breadfruit* OR broccoli* OR “brussels sprout*” OR cantaloupe* OR caper* OR carrot* OR cauliflower* OR celeriac* OR celery OR celeries OR chard* OR cherr* OR “chick pea*” OR “Chinese cabbage*” OR chive* OR citron* OR citrus OR coconut* OR “collard greens” OR crabapple* OR cranberr* OR cress* OR cucumber* OR currant* OR daikon* OR “dandelion green*” OR date OR dates OR “dragon fruit*” OR durian* OR “edible plant*” OR eggplant* OR elderberr* OR endive* OR “fava bean*” OR fennel* OR fig OR figs OR fruit* OR garlic* OR ginger* OR gourd* OR grape* OR “green bean*” OR greens OR guava* OR honeydew* OR “hot chili pepper*” OR “iceberg lettuce*” OR jackfruit* OR jicama* OR kiwi* OR kumquat* OR leek* OR legume* OR lemon* OR lentil* OR lettuce* OR “lima bean*” OR lime OR limes OR lingonberr* OR loquat* OR lychee* OR maize* OR “mandarin orange*” OR mango* OR marionberr* OR melon* OR mulberr* OR “mung bean*” OR mushroom* OR “mustard green*” OR nectarine* OR nut OR nuts OR okra* OR olive* OR onion* OR orange* OR papaya* OR parsley* OR parsnip* OR “passion fruit*” OR “pattypan squash*” OR pea OR peas OR peach* OR peanut* OR peapod* OR pear* OR pepper* OR persimmon* OR pickle* OR pineapple* OR plantain* OR plum* OR pluot* OR pomegranate* OR pomelo* OR potato* OR prune* OR pumpkin* OR quince* OR radicchio* OR radish* OR raisin* OR raspberr* OR rhubarb* OR rocket* OR romaine* OR rutabaga* OR salad* OR salsa* OR salsif* OR scallion* OR seaweed* OR shallot* OR sorrel* OR soybean* OR spinach* OR sprouts OR spuds OR squash OR squashes OR “star fruit*” OR strawberr* OR “string bean*” OR succotash* OR “sweet potato*” OR “Swiss chard*” OR tangelo* OR tangerine* OR taro* OR tomatillo* OR tomato* OR tuber OR tubers OR turnip* OR “ugli fruit*” OR vegetable* OR wasabi* OR “water chestnut*” OR watercress* OR watermelon* OR yam OR yams OR zucchini*).ti,ab.

1,878,056 results

3

(“South East Asia*” OR “Southeast Asia*” OR “Southeastern Asia*” OR Brunei* OR Cambodia* OR “East Timor” OR Indonesia* OR Laos OR Malaysia* OR Myanmar* OR Burma OR Burmese OR Philippine* OR Singapore* OR Thailand OR Thai OR Vietnam* OR “Viet Nam” OR Andaman* OR Nicobar* OR “Christmas Island” OR “Cocos Islands” OR “East Asia*” OR “Eastern Asia*” OR China OR Chinese OR “Hong Kong” OR Macau OR Mongolia* OR Korea* OR Japan* OR Taiwan*).ti,ab.

651,739 results

4

1 AND 2 AND 3

1759 results

5 limit 4 to English language

1267. Results
