# Supplementary material for: The Food Environments of Fruit and Vegetable Consumption in East and Southeast Asia: A Systematic Review
Source: Nutrients. 2021 Jan 4;13(1):148. doi: 10.3390/nu13010148 (PMC7824134; doi:10.3390/nu13010148)
Supplement: Supplementary file 1 [file nutrients-13-00148-s001.pdf]

SUPPLEMENTARY DATA.

Table S1. Journals, Citations No., H-Index, and Quality Evaluation Scores.

ID	Journals	Citations No.	H-Index	QE1	QE2	QE3	QE4	QE5	Scores
1	IH	1	27	2	2	2	1	1	8
2	AJARE	1	46	2	2	2	1	1	8
3	BFJ	49	74	2	1	2	2	1	8
4	FC	27	114	2	2	2	2	1	9
5	CJAE	36	35	2	2	2	1.5	1	8.5
6	AMJ	7	32	2	1	2	1	1	7
7	JFAKU	33	16	2	1	2	1	1	7
8	EE	5	53	2	1	2	1.5	1	7.5
9	FP	7	95	2	1	2	2	1	8
10	BFJ	36	74	2	2	2	2	1	9
11	NRP	9	29	2	2	2	1	1	8
12	BFJ	2	74	2	2	2	1.5	1	8.5
13	IJISSS	0	11	2	2	2	1	1	8
14	BJN	31	178	2	1	2	2	0	7
15	FS	1	41	2	2	2	1	1	8
16	PO	6	300	2	2	2	2	1	9
17	FQP	38	110	2	2	2	2	1	9
18	STE	10	224	2	1	2	2	1	8
19	IJBNPA	2	104	2	1	2	2	1	8
20	EHPM	28	34	2	2	2	1.5	0	7.5
21	JCM	93	91	2	2	2	2	1	9
22	EB	0	27	2	1	2	1	1	7
23	JFR	11	26	2	1	2	1	1	7
24	IJER	3	19	2	1	2	1	1	7
25	JCP	62	173	2	2	2	2	1	9
26	BFJ	13	74	2	1	2	1.5	1	7.5
27	CAER	2	19	2	1	2	1	1	7
28	JRS	41	96	2	1	2	2	1	8

Note. AB-Agribusiness, AJARE-Australian Journal of Agricultural and Resource Economics, APJCN-Asia Pacific Journal of Clinical Nutrition, AMJ-Australasian Marketing Journal, BFJ-British Food Journal, BJN-British Journal of Nutrition, CAER-China Agricultural Economic Review, CJAE-Canadian Journal of Agricultural Economics, EB-Economics Bulletin, EE-Empirical Economics, EHPM-Environmental Health and Preventive Medicine, FC-Food Control, FP-Food Policy, FQP-Food Quality and Preference, FS-Food Security, IH-International Health, IJBNPA-International Journal of Behavioral Nutrition and Physical Activity, IJER-International Journal of Economic Research, IJISSS-International Journal of Information Systems in the Service Sector, JCM-Journal of Consumer Marketing, JCP-Journal of Cleaner Production, JFAKU-Journal of the Faculty of Agriculture Kyushu University, JFR-Journal of Food Research, JRS-Journal of Rural Studies, NRP-Nutrition Research and Practice, PO-PLoS ONE, STE-Science of the Total Environment.

Table S1. *Cont.*

ID	Journals	Citations No.	H-Index	QE1	QE2	QE3	QE4	QE5	Scores
29	FP	43	95	2	2	2	2	1	9
30	APJCN	8	72	2	2	2	1.5	1	8.5
31	AB	5	42	2	1	2	1	1	7

Note. AB-Agribusiness, AJARE-Australian Journal of Agricultural and Resource Economics, APJCN-Asia Pacific Journal of Clinical Nutrition, AMJ-Australasian Marketing Journal, BFJ-British Food Journal, BJN-British Journal of Nutrition, CAER-China Agricultural Economic Review, CJAE-Canadian Journal of Agricultural Economics, EB-Economics Bulletin, EE-Empirical Economics, EHPM-Environmental Health and Preventive Medicine, FC-Food Control, FP-Food Policy, FQP-Food Quality and Preference, FS-Food Security, IH-International Health, IJBNPA-International Journal of Behavioral Nutrition and Physical Activity, IJER-International Journal of Economic Research, IJISSS-International Journal of Information Systems in the Service Sector, JCM-Journal of Consumer Marketing, JCP-Journal of Cleaner Production, JFAKU-Journal of the Faculty of Agriculture Kyushu University, JFR-Journal of Food Research, JRS-Journal of Rural Studies, NRP-Nutrition Research and Practice, PO-PLoS ONE, STE-Science of the Total Environment.
